# A highly efficient and faithful MDS patient-derived xenotransplantation model for pre-clinical studies

**DOI:** 10.1038/s41467-018-08166-x

**Published:** 2019-01-21

**Authors:** Yuanbin Song, Anthony Rongvaux, Ashley Taylor, Tingting Jiang, Toma Tebaldi, Kunthavai Balasubramanian, Arun Bagale, Yunus Kasim Terzi, Rana Gbyli, Xiaman Wang, Xiaoying Fu, Yimeng Gao, Jun Zhao, Nikolai Podoltsev, Mina Xu, Natalia Neparidze, Ellice Wong, Richard Torres, Emanuela M. Bruscia, Yuval Kluger, Markus G. Manz, Richard A. Flavell, Stephanie Halene

**Affiliations:** 1 Section of Hematology, Department of Internal Medicine and Yale Comprehensive Cancer Center, Yale University School of Medicine, New Haven, CT USA; 2 Department of Immunobiology, Yale University School of Medicine, New Haven, CT USA; 3 Fred Hutchinson Cancer Research Center, Program in Immunology, Clinical Research Division, and Department of Immunology, University of Washington School of Medicine, Seattle, WA USA; 4 Department of Pathology, Yale University School of Medicine, New Haven, CT USA; 5 Laboratory of Translational Genomics, Centre for Integrative Biology (CIBIO), University of Trento, Trento, Italy; 6 University of New Haven, New Haven, CT USA; 7 Department of Medical Genetics, Faculty of Medicine, Baskent University, Ankara, Turkey; 8 Department of Hematology, The Second Affiliated Hospital of Xi’an Jiaotong University, Xi’an, People’s Republic of China; 9 Department of Laboratory Medicine, Shenzhen Children’s Hospital, Shenzhen, People’s Republic of China; 10 Section of Hematology/Oncology, VA Medical Center, West Haven, CT USA; 11 Department of Laboratory Medicine, Yale University School of Medicine, New Haven, CT USA; 12 Department of Pediatrics, Yale University School of Medicine, New Haven, CT USA; 13 Interdepartmental Program in Computational Biology and Bioinformatics, Yale University, New Haven, CT USA; 14 Program of Applied Mathematics, Yale University, New Haven, CT USA; 15 Hematology, University Hospital and University of Zurich, Zurich, Switzerland; 16 Howard Hughes Medical Institute, Yale University, New Haven, CT USA

## Abstract

Comprehensive preclinical studies of Myelodysplastic Syndromes (MDS) have been elusive due to limited ability of MDS stem cells to engraft current immunodeficient murine hosts. Here we report a MDS patient-derived xenotransplantation model in cytokine-humanized immunodeficient “MISTRG” mice that provides efficient and faithful disease representation across all MDS subtypes. MISTRG MDS patient-derived xenografts (PDX) reproduce patients’ dysplastic morphology with multi-lineage representation, including erythro- and megakaryopoiesis. MISTRG MDS-PDX replicate the original sample’s genetic complexity and can be propagated via serial transplantation. MISTRG MDS-PDX demonstrate the cytotoxic and differentiation potential of targeted therapeutics providing superior readouts of drug mechanism of action and therapeutic efficacy. Physiologic humanization of the hematopoietic stem cell niche proves critical to MDS stem cell propagation and function in vivo. The MISTRG MDS-PDX model opens novel avenues of research and long-awaited opportunities in MDS research.

## Abstract (web summary)

Myelodyplastic hematopoietic stem cells (MDS HSC) have eluded in vivo modeling. Here the authors present a highly efficient MDS patient-derived xenotransplantation model in cytokine-humanized mice with replication of the donors’ genetic complexity and myeloid, erythroid, and megakaryocytic lineage dysplasia.

## Introduction

Myelodysplastic syndrome (MDS) is a group of heterogeneous disorders of the hematopoietic stem cell characterized by recurrent genetic aberrations in genes of essential pathways, including transcription factors, epigenetic regulators, cohesin complex genes, DNA repair genes, and key factors of the spliceosome (see refs. ^1,2^ and reviewed in ref. ^3^).

Long-term hematopoietic stem cells (HSCs) cannot be expanded in culture and only rare MDS cell lines exist^4–6^, creating an unmet need for in vivo models of primary MDS. Xenotransplantation of primary human MDS stem cells into currently available immunodeficient mice, such as NOD*-scid Il2rg*^*−/−*^ (NSG), has demonstrated limited success with low efficiency and transient engraftment, skewing towards the lymphoid lineage, and engraftment mostly restricted to the injected tibial bone when aided by co-injection of human mesenchymal stem cells (MSCs)^7–10^. Human cytokines provided by constitutive, transgene-driven expression in the NSG-SGM3 model (overexpressing human stem cell factor (SCF), granulocyte-monocyte-colony-stimulating factor (GM-CSF), and interleukin-3 (IL3) from a cytomegalovirus promoter), improve myeloid differentiation and cellular proliferation, yet stem cell maintenance is impaired^11–15^. This limitation is overcome transiently by co-injection of autologous human MSCs^16^ or by creation of an ossicle from human MSCs that provides an improved human stem cell environment^17^. These latter two approaches have limited applicability in pre-clinical studies that require a highly efficient, high-throughput approach.

We here present a novel highly efficient MDS xenotransplantation model, in humanized immunodeficient “MISTRG” mice, expressing humanized M-CSF, IL3/GM-CSF, SIRP alpha, and Thrombopoietin in the Rag^*−*/*−*^, IL2Rγ^*−*/*−*^ genetic background from their endogenous murine loci. MISTRG mice have previously been shown to be highly permissive for human hematopoiesis and support robust reconstitution of human lymphoid and myelo-monocytic cellular systems^18,19^. We demonstrate that primary healthy bone marrow- (BM) and MDS BM-derived CD34^+^ cells from lower-risk (International Prognostic Scoring System (IPSS) low- and intermediate 1) and higher-risk (intermediate 2 and high) MDS, defined by the number of cytopenias, blast percentage in BM, and cytogenetic abnormalities, efficiently engraft in MISTRG mice and give rise to multi-lineage hematopoiesis and specifically to myelo-, erythro-, and mekagaryopoiesis. We demonstrate that MDS patient-derived MISTRG xenotransplants (MDS MISTRG PDX) support the MDS stem cell across all MDS subtypes, replicate the patients’ MDS immunophenotype and dysplastic features, faithfully reproduce the clonal complexity of the disease at time of diagnosis and along disease progression, and are ideally suited for the testing of targeted therapeutics. Thus, given the high multi-lineage engraftment efficiency for normal and MDS HSCs and the histologic and clonal fidelity, MISTRG PDX represent a significant advancement over currently available xenotransplantation models and an ideal in vivo pre-clinical model for MDS.

## Results

### MISTRG engraft healthy adult bone marrow-derived CD34^+^ HSPCs

**Fig. 1:**
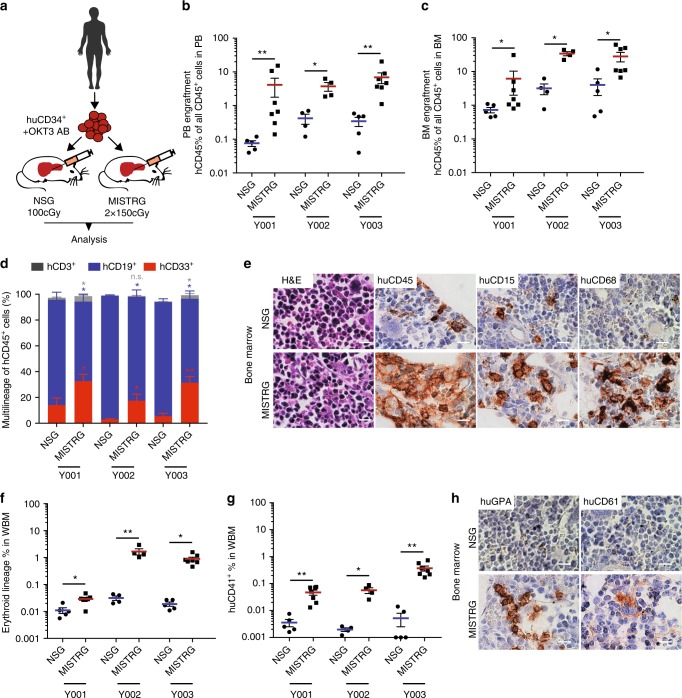
Enhanced engraftment of adult healthy bone marrow (BM)-derived CD34^+^ hematopoietic stem and progenitor cells (HSPCs) in human cytokine-knockin MISTRG mice. **a** Universal experimental setup. Human BM-derived CD34^+^ HSPCs were pre-incubated with anti-CD3 antibody (OKT3) and injected intrahepatically into newborn (D2–3) NSG or MISTRG mice conditioned with the respective maximum tolerated irradiation doses (NSG 100 cGy, MISTRG 2 × 150 cGy). Mice were analyzed 10–17 (healthy BM), 13–30 (myelodysplastic syndrome (MDS)), and 9−24 (acute myeloid leukemia (AML)) weeks post transplantation. **b**, **c** Comparison of overall human CD45^+^ engraftment in peripheral blood (PB) and BM in NSG versus MISTRG mice. Individual mice are represented by symbols. **d** Relative distribution of myeloid CD33^+^ (red), B-lymphoid CD19^+^ (blue), and T-lymphoid CD3^+^ (gray) cells as % of human CD45^+^ cells in NSG vs. MISTRG mice. **e** BM histology of representative NSG and MISTRG mice from (**d**). Hematoxylin and eosin (H&E) and immunohistochemistry (IHC) stains for huCD45, huCD15, huCD68 in NSG (top) and MISTRG BM (bottom row) (scale bars 10 µm, original magnification 60×). **f**, **g** Comparison of erythroid and megakaryocytic lineage engraftment in BM of NSG and MISTRG mice. **h** BM histology of representative NSG and MISTRG mice from (**d**). H&E and IHC stains for huCD235 and huCD61 as in (**e**). For detailed sample information see Supplementary Table 1. In (**c**, **d**, **e**, **f**, **g**) data are represented as means ± S.E.M.; Mann–Whitney test: n.s. not significant, *p < 0.05, **p < 0.01

Adult CD34^+^ hematopoietic stem and progenitor cells (HSPCs) engraft with significantly lower efficiency in immunodeficient mice compared to human fetal liver- or cord blood-derived CD34^+^ cells^18^. However, the majority of myeloid malignancies and in particular MDS occur in the aging adult with quantitative and qualitative limitations to the stem cell population of interest. We transplanted healthy BM-derived CD34^+^ cells from adult patients, in whom BM involvement by their underlying disease was excluded (see Supplementary Table 1), intrahepatically into newborn NSG and MISTRG mice irradiated with maximum tolerated doses for each strain (Fig. 1a)^18^. The maximum tolerated radiation in NSG mice is limited due to the inherent DNA repair defect conferred by the *scid* mutation^20,21^. Samples were CD34 enriched or CD3 depleted (Supplementary Figure 1a), and further purged of mature T cells by pre-treatment with the humanized anti-CD3 antibody OKT3 for prevention of graft versus host disease^22^. Highest available rather than a fixed cell number were injected as equal split-donor grafts into NSG and MISTRG mice to maximize engraftment for each primary sample.

Analysis consisting of complete blood counts and histology (representative subset), and flow cytometry of peripheral blood (PB), BM, and spleen, was performed at least 12 weeks post transplantation, with >85% survival for both NSG and MISTRG recipient mice to planned analysis (Supplementary Figure 1b). Flow cytometric analysis consisted of assessment of overall human leukocyte engraftment (huCD45) as a function of all (murine and human) leukocytes as well as assessment for human erythroid and megakaryocytic engraftment within the murine and human CD45 negative fraction. Erythroid and megakaryocytic lineage engraftment based on CD45 negativity and high transferrin receptor (huCD71)/glycophorin A (huCD235) or huCD41 expression, respectively, were quantitated as % of all single live cells in whole BM (Supplementary Figure 1c).

MISTRG mice show significantly higher huCD45^+^ engraftment in PB and BM than NSG mice (Fig. 1b, c) and support enhanced differentiation towards myelopoiesis (Fig. 1d) over lymphopoiesis, rectifying a key difference between human and mouse hematopoiesis. CD3^+^ T cells are efficiently depleted with OKT3 treatment of the graft and represent only a minor fraction. Histologically, myeloid cells express the mature myeloid markers huCD15 and huCD68. As previously described^18,23^, expression of human GM-CSF and macrophage colony-stimulating factor (M-CSF) enhance myeloid maturation with differentiation towards mature granulocytes and macrophages (Fig. 1e and Supplementary Figure 1d) with repopulation of bone marrow as well as spleen and non-hematopoietic tissues, such as liver (Supplementary Figure 1e).

Interestingly, MISTRG bone marrows show significantly higher numbers of erythroid progenitor cells (CD71^bright^, GPA^+^) (Fig. 1f, h) as well as human CD41+ megakaryocytes and platelets (Fig. 1g, h).

In summary, MISTRG mice support superior healthy adult BM xenografts with tri-lineage representation.

### MISTRG efficiently support all risk MDS PDX with multi-lineage output

NSG mice have represented a major breakthrough in xenotransplantation studies due to the lack of mature murine T, B, and functional natural killer (NK) cells^24^ and the presence of the *Sirp*α gene polymorphism, allowing enhanced binding of the mSirpα to human CD47^25,26^. However, engraftment of MDS BM-derived CD34^+^ HSPCs remains a challenge, despite several alterations to NSG mice and the transplantation protocol^7–10,12–14,16^. We engrafted MDS CD34^+^ (or CD3-depleted) BM cells into NSG and MISTRG recipients as split-donor grafts, as in Fig. 1a. To avoid a priori exclusion of lower-risk MDS samples or patient samples with low cell numbers, CD34^+^ cell injections for different samples ranged from 0.5 × 10^5^ to 1 × 10^6^ cells per recipient mouse, while maintaining the same cell number for all recipients within each experiment (for detailed patient and sample information see Supplementary Table 1).

**Fig. 2:**
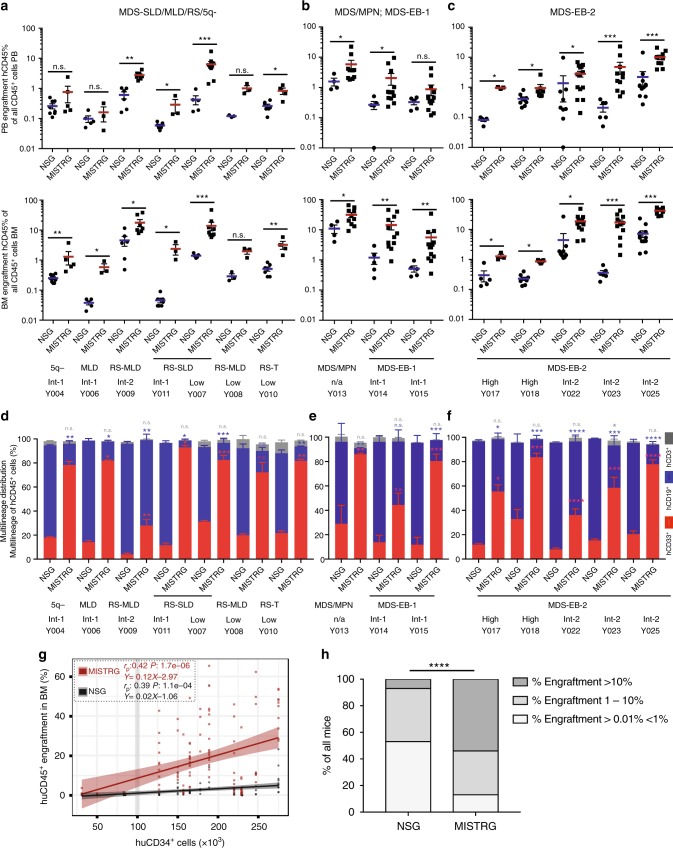
Enhanced engraftment of lower- and higher-risk myelodysplastic syndrome (MDS) in MISTRG mice. **a**–**c** Analysis of huCD45 engraftment was performed as detailed in Fig. 1a at >12 weeks post transplantation. **a** Analysis of MDS-5q-, -SLD-, -MLD-, and -MLD-RS-engrafted NSG and MISTRG mice. **b** Analysis of MDS/MPN and MDS-EB-1-engrafted NSG and MISTRG mice. **c** Analysis of MDS-EB-2-engrafted NSG and MISTRG mice. MISTRG afford significantly higher engraftment than NSG in lower- and higher-grade MDS. **d**–**f** Relative distribution of myeloid CD33^+^ (red), B-lymphoid CD19^+^ (blue), and T-lymphoid CD3^+^ (gray) cells as % of human CD45^+^ cells in NSG vs. MISTRG mice. Stacked bar graphs represent means ± S.E.M. Mann–Whitney test; n.s. not significant, *p < 0.05, **p < 0.01, ***p < 0.001, ****p < 0.0001. **g** Split-donor huCD45^+^ BM engraftment in NSG (black) versus MISTRG (red) mice plotted against CD34^+^ cell number injected/mouse. Individual mice are represented by symbols. Linear regression, Pearson's correlations and *p* values of % engraftment to CD34^+^ cell number in NSG (*r* = 0.39, *p* < 0.0001) vs. MISTRG (*r* = 0.42, *p* < 0.0001) are displayed. **h** Percentage of transplanted mice with huCD45^+^ bonemarrow (BM) engraftment levels >0.01% < 1%, 1–10%, and >10% for split-donor grafts in NSG (59/111, 44/111, and 8/111, respectively) and MISTRG (20/154, 51/154, and 83/154, respectively) mice (Fisher’s exact test, ****p < 0.0001 for NSG vs. MISTRG). For detailed patient sample information see Supplementary Table 1. SLD single lineage dysplasia; MLD multi lineage dysplasia; RS Ringsideroblasts; MPN myeloproliferative neoplasm

We engrafted a total of 10 low- and intermediate 1 risk and 8 intermediate 2 and high-risk MDS samples (Fig. 2 and Supplementary Figure 2). MISTRG consistently resulted in higher engraftment than NSG for all MDS subtypes in peripheral blood (top row) and bone marrow (bottom row) (Fig. 2a–c). Only 2 out of 29 samples (MDS with multi-lineage dysplasia (MLD) Y006, MDS with excess blasts 2 (EB-2) Y018), injected at <1 × 10^5^ CD34^+^ cells/mouse displayed BM engraftment levels <1% in MISTRG. The engraftment persisted until the time of analysis, >12 weeks post transplantation, without development of compromising anemia or thrombocytopenia in recipient mice (Supplementary Figure 3a-c) or differences in survival between MISTRG and NSG mice (Supplementary Fig 1b), interestingly with similar engraftment in female and male mice of the respective strains (Supplementary Figure 3d), not seen with engraftment in adult NSG mice in previous studies^27^. As described previously for normal hematopoiesis, CD34^+^ cells from MDS bone marrow give rise to myeloid predominant grafts, while NSG mice give rise to lymphoid-predominant grafts (Fig. 2d–f). Expression of human M-CSF, GM-CSF, and IL-3 further enhances maturation of MDS-derived myeloid cells with differentiation profiles close to the patients’ phenotypes (representative example given in Supplementary Figure 2a).

When plotting engraftment in all mice against injected CD34^+^ cell number, it is evident that a minimum number of 1 × 10^5^ CD34^+^ cells/mouse was required for reliable engraftment (Fig. 2g). Interestingly, increasing cell numbers resulted in improved engraftment in MISTRG while engraftment in NSG recipients remained limited. Although all recipients engrafted above 0.01%, the minimum engraftment threshold set in several studies, for the purpose of pre-clinical modeling a higher engraftment threshold may prove advantageous. When comparing all split-donor graphs, engraftment of >1% was achieved in 85% of MISTRG and in 52% of NSG mice. Importantly, engraftment levels of >10%, more likely to reliably afford pre-clinical studies, were achieved in 53% of MISTRG but in less than 10% of NSG mice (Fig. 2h).

**Fig. 3:**
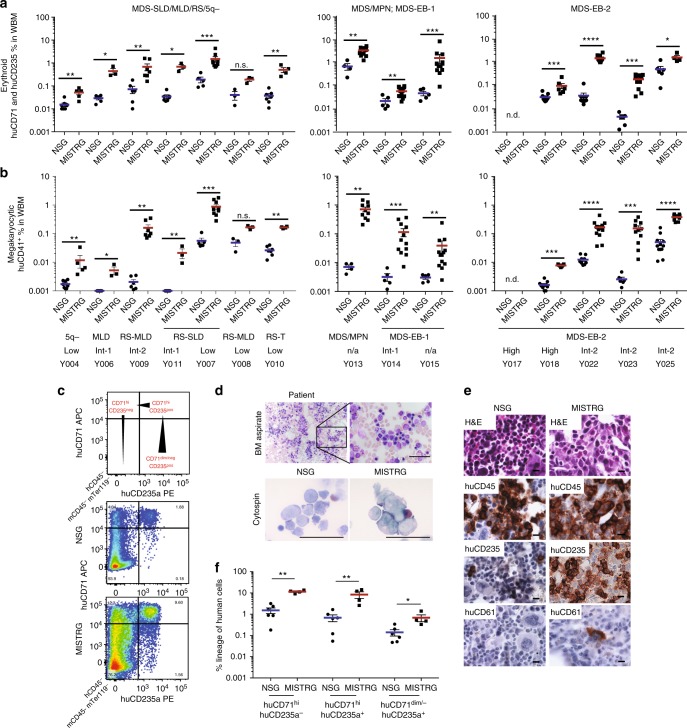
Erythroid and megakaryocytic lineage representation in myelodysplastic syndrome (MDS) MISTRG xenografts. **a** Analysis of human erythroid lineage output in NSG versus MISTRG mice engrafted with lower- and higher-risk MDS (as in Fig. 2) via determination of CD71^+/*−*^ huCD235^*−*/+^ expression in hu/muCD45^*−*^ muTer119^*−*^ bone marrow (BM). **b** Analysis of human megakaryocytic lineage output (MK and platelets) in NSG versus MISTRG mice (engrafted as in Fig. 2) via determination of huCD41^+^ in hu/muCD45^*−*^ BM. **c** NSG and MISTRG xenografted with MDS-EB-2 (Y025) BM with inverted myeloid/erythroid ratio. **d** Patient BM aspirate (top) and sorted human erythroblasts from engrafted NSG and MISTRG BM (bottom) (for overall engraftment see Fig. 2c, Y025). **e** Representative BM histology from representative NSG and MISTRG recipients engrafted >1% stained with hematoxylin and eosin (H&E), huCD45, huCD235, and huCD61. **f** Representative fluorescence-activated cell sorting (FACS) plots of erythroid lineage differentiation based on huCD71 and huCD235 expression in huCD45^*−*^ muCD45^*−*^ mTer119^*−*^ cells (huCD71^hi^huCD235a^−^ (pro-erythroblasts (EB)), huCD71^hi^huCD235a^+^ (basophilic EB/normoblasts), huCD71^−^huCD235a^+^ (reticulocytes, RBC))

Importantly, we here show for the first-time engraftment of primary adult MDS-derived erythropoiesis and megakaryopoiesis. Analysis of the CD45^neg^ population (Supplementary Figure 1c) revealed significant contribution by human erythropoiesis (defined by huCD71^bright^ and huCD235 positivity among CD45^neg^ cells) and megakaryopoiesis (huCD41^+^ among CD45^neg^ cells) in immunodeficient mice, with significantly higher representation in MISTRG mice for all subtypes of MDS (Fig. 3a, b). CD3 depletion of primary MDS BM samples, similar to CD34 enrichment, resulted in similar engraftment levels in PB and BM (Supplementary Figure 2,b, c), myeloid predominant grafts in MISTRG mice (Supplementary Figure 2d), and significant erythropoietic and megakaryocytic development (Supplementary Figure 2e, f).

Importantly, MISTRG mice revealed erythroid differentiation as evident by progressive acquisition of glycophorin A expression and downregulation of transferrin receptor expression with maturation. As an example, a patient’s BM aspirate (MDS-EB-2, Y025) with significant erythroid hyperplasia (Fig. 3d, top) is shown. Cytospins of sorted erythroblasts of engrafted primary MISTRG and to a lesser extent of engrafted NSG revealed erythroid precursors with signs of dysplasia, such as binuclear forms (Fig. 3d, bottom). Importantly, BM histology revealed prominent development of huCD235^+^ erythroid progenitors in MISTRG mice (Fig. 3e), confirmed by flow cytometric determination of huCD71^pos^ and huCD235^pos^ (gated on CD45^neg^, mTer119^neg^ and huCD45^neg^ cells) erythroid development as shown in Fig. 3f with limited erythroid development in NSG mice. This significant support of erythropoiesis in MISTRG mice is not unique to MDS, but also evident in xenografts from healthy BM- (Supplementary Figure 4a) and human umbilical cord blood-derived CD34+ HSCPs (Supplementary Figure 4b–f). Importantly, erythroid lineage representation is present in secondary MDS xenograft recipients (Supplementary Figure 4g–i), suggesting that it is derived from the MDS stem cell.

To assure that xenografts are derived from the malignant MDS clone we performed mutational analysis by targeted exome sequencing of patient samples and corresponding murine cell-depleted patient-derived xenografts. Presence of corresponding driver mutations at equivalent variant allele frequencies (VAFs) confirmed engraftment of MDS-derived hematopoiesis (Supplementary Table 2).

In summary, MISTRG mice support superior long-term engraftment of clonal MDS with representation of mature myeloid lineages and importantly MDS-derived erythro- and megakaryopoiesis.

### MISTRG replicate MDS heterogeneity and myeloid dysplasia and clonal evolution

Although several murine models of MDS have been generated, the finding of dysplasia is rare and frequently subtle (reviewed in ref. ^28^). Currently available xenotransplantation models have not been shown to replicate myelodysplasia, the essential diagnostic criterion for MDS, nor to support development of erythro- and megakaryopoiesis, two of the three principal cell lines affected in MDS^29,30^.

Mutations in the RNA splicing factor SF3B1 are pathognomonic for MDS with ring sideroblasts (RS). To date, no model exists that allows studying the development of RS. *Sf3b1* mutant mice do not develop RS^31^ and despite successful protocols for erythroid differentiation in vitro, development of RS has not been described.

**Fig. 4:**
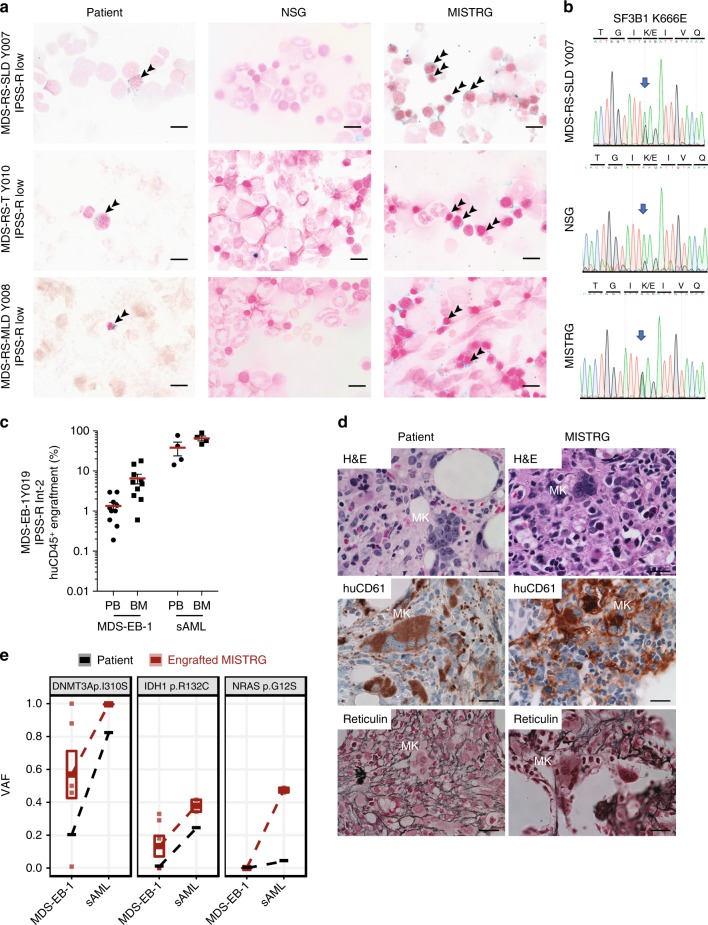
MISTRG replicate myelodysplasia and clonal evolution upon disease progression. **a** NSG and MISTRG were engrafted with low and int-1 risk *Sf3B1* mutant myelodysplastic syndrome (MDS) with ring sideroblasts (see Figs. 2, 3) and patient and NSG and MISTRG xenografts were stained with Prussian blue iron stain (scale bars 10 µm, original magnification 60×). **b** *SF3B1* mutation was verified in the patient’s and representative NSG and MISTRG xenografts by Sanger sequencing. **c**–**e** MISTRG engrafted with consecutive MDS-EB-1 and secondary acute myeloid leukemia (sAML) samples from the same patient (Y019 and Y028, respectively). **c** Overall (huCD45^+^) engraftment in peripheral blood (PB) and bone marrow (BM). Individual mice are represented by symbols, with means ± S.E.M. **d** Histology from MDS-EB-1 (Y019) diagnostic BM and representative engrafted MISTRG BM. Hematoxylin and eosin (H&E) and huCD61 stains reveal human megakaryocytic dysplasia and reticulin stain reveals bone marrow fibrosis (high-power magnification scale bars 20 µm). **e** Targeted exome sequencing results from MISTRG xenografted with same patient’s primary MDS-EB-1 diagnosis samples and sAML at the time of disease progression. For each mutation, variant allele frequencies (VAFs) are shown for the patient (black) and representative MISTRG (red) mice with engraftment levels >1%. Mean VAF values between MDS-EB-1 and sAML are connected by lines

We engrafted three low-risk MDS samples with RS with single and multi lineage dysplasia(RS-SLD, RS-MLD) and  with thrombocytosis (RS-T)) to evaluate dysplasia in human MDS xenografts. All three patient samples successfully engrafted in MISTRG mice (Fig. 2a) with development of erythropoiesis (Fig. 3a). Iron stain of patient bone marrow and MISTRG xenografts, but not of NSG xenografts, revealed RS (Fig. 4a). Sanger sequencing of the patient’s BM DNA as well as DNA from engrafted NSG and MISTRG recipient mice confirmed presence of the *SF3B1* K666E mutation and engraftment of the mutant MDS clone.

To determine whether MISTRG MDS xenografts could replicate megakaryocytic dysplasia, we engrafted a sample with marked MK dysplasia. MISTRG mice efficiently engrafted with sample Y019 (MDS-EB-1 with normal karyotype, Fig. 4c, left) displayed numerous dysplastic megakaryocytes and reticulin fibrosis, faithfully replicating the patient’s MDS dysplastic features (Fig. 4d). MISTRG mice engrafted with the same patient’s secondary acute myeloid leukemia (sAML) sample obtained at the time of disease progression (Fig. 4c; Y028, sAML, NK) did not show these features. Targeted exome sequencing of the MDS xenografts confirmed derivation from the patient’s DNMT3a-mutant MDS clone (Fig. 4e). Interestingly, an isocitrate dehydrogenase 1 (IDH1) mutation was identified in several of the MISTRG mice (VAF 18–32%) engrafted with the patient’s initial MDS diagnosis sample (MDS-EB-1, Fig. 4e). This IDH1 mutation was not reported in the patient at the time of MDS diagnosis, but present at the time of disease progression to sAML (sAML, VAF 24%, Fig. 4e). Re-sequencing detected the IDH1 R132C mutation in the MDS diagnosis sample at a VAF of ~1% (Fig. 4e, middle panel, Supplementary Tables 1 and 2). Interestingly, in the sAML engrafted MISTRG mice, a new NRAS G12S mutation defined the dominant clone, again detectable in the patient’s sAML at a VAF <5% in addition to the dominant DNMT3a and IDH1 mutations. RAS mutations have been described as a potential mechanism of resistance to mutant IDH inhibitor treatment^32^ and identification of these mutant clones in a pre-clinical MDS PDX may thus guide the use of pre-emptive combination regimens.

In addition to erythroid and megakaryocytic dysplasia, we noted a functional difference of myeloid cells in normal BM, MDS, and AML engrafted MISTRG mice (Supplementary Figure 4j). While healthy BM xenografts engraft BM, liver, and spleen and give rise to resident tissue macrophages in all three tissues (Supplementary Figure 5a), in age-matched patient-derived MDS xenografts these populations are mostly absent from the spleen and the liver, consistent with a functional defect of the myeloid lineages in MDS (Supplementary Figure 5b). This is in stark contrast to myeloid leukemia, where immature blasts infiltrate spleen and liver (Supplementary Figure 5c).

In summary, we here present the first MDS PDX model that replicates myelodysplasia and that affords the study of MDS erythropoietic and megakaryopoietic defects. In addition, we show that MISTRG MDS PDXs may predict clonal evolution upon disease progression.

### The MISTRG humanized niche allows propagation of MDS HSCs via serial transplantation

HSCs are critically dependent on the stem cell niche. MDS HSCs are dysfunctional and their in vitro and in vivo propagation has been elusive to date. We hypothesized that cytokine humanization of the HSC niche would afford homing and engraftment of primary patient-derived MDS long-term HSCs in MISTRG mice capable of serial repopulation. Human thrombopoietin is essential for stem cell function^33,34^. IL3, GM-CSF, or M-CSF are not directly implicated in stem cell maintenance, but via their role in myeloid cell development, such as BM macrophages, they may indirectly supply additional niche signals^35,36^. We assessed human versus murine cytokine expression in MDS (Supplementary Figure 6a) and murine MISTRG and NSG BM-derived mesenchymal stromal cell (MSC) cultures (Supplementary Figure 6b). Human and MISTRG MSCs but not NSG MSCs express human THPO, GM-CSF, and M-CSF instead of their murine counterparts (Supplementary Figure 6c) at physiologic levels similar to human MSCs (Supplementary Figure 6d). IL3, as expected, is not expressed in MSCs^37^.

**Fig. 5:**
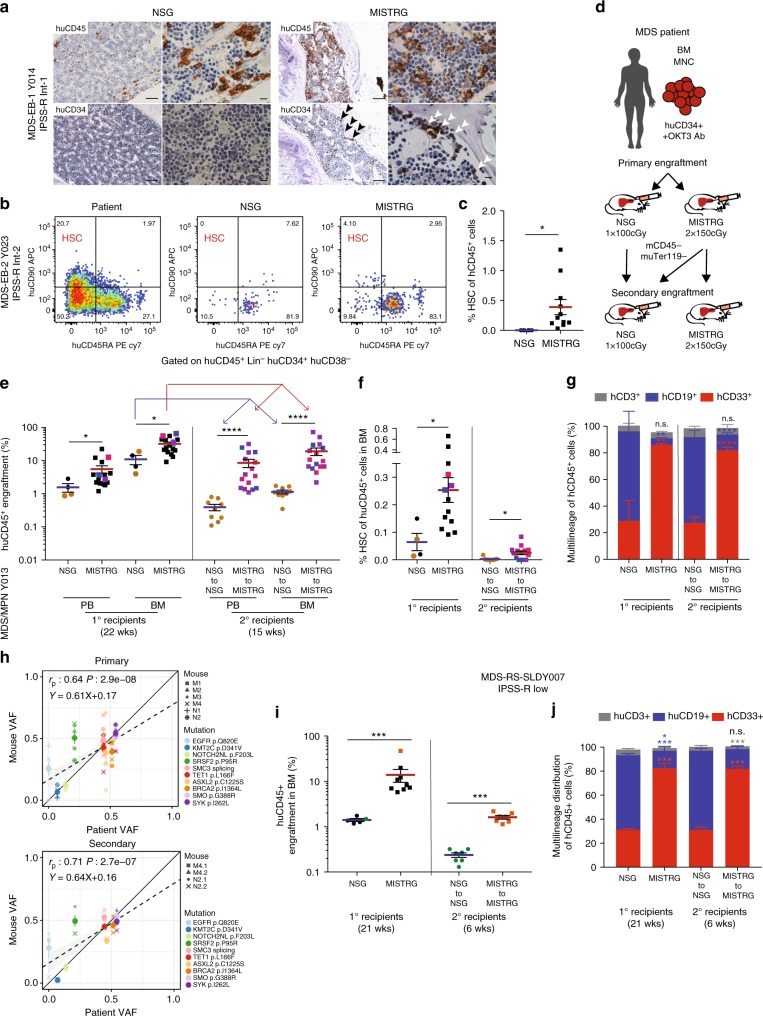
MISTRG support phenotypic and functional clonal myelodysplastic syndrome (MDS) stem cells with long-term multi-lineage engraftment potential in serial transplantation. **a** Representative immunohistochemistry (IHC) for huCD45 and huCD34 distribution in NSG (of *n* = 5) and MISTRG (of *n* = 12) bone marrow (BM) engrafted with MDS-EB-1 (Y014; scale bars for low-power field: 100 µm, original magnification 10×; high-power field: 10 µm, original magnification 60×). **b**, **c** MISTRG engraft phenotypic MDS stem cells. **b** Representative fluorescence-activated cell sorting (FACS) plots and **c** quantification of hematopoietic stem cell (HSC) representation (lin^−^CD34^+^CD38^−^CD45RA^−^CD90^+^ of huCD45^+^) of corresponding patient (high-risk MDS-EB2, Y023), and NSG and MISTRG xenografts. **d**–**j** MISTRG engraft functional MDS stem cells. **d** Secondary xenotransplantation experimental setup. **e**–**h** Primary and secondary transplantation of MPN/MDS sample with 3% blasts (Y013) comparing **e** overall huCD45^+^ engraftment in peripheral blood (PB) and BM, **f** phenotypic HSC % in BM, and **g** relative distribution of myeloid CD33^+^ (red), B-lymphoid CD19^+^ (blue), and T-lymphoid CD3^+^ (gray) cells as % of human CD45^+^ cells in NSG vs. MISTRG mice. In scatter plots individual mice are represented by symbols with means ± S.E.M.; symbols for corresponding 1° and 2° recipient mice are color coded; statistics represent Mann–Whitney test; n.s. not significant, *p < 0.05, **p < 0.01, ***p < 0.001, ****p < 0.0001. Stacked bar graphs represent means ± S.E.M. Mann–Whitney test; n.s. not significant, *p < 0.05, **p < 0.01, ***p < 0.001, ****p < 0.0001. **h** Clonality was determined in representative primary and secondary MISTRG recipients with engraftment levels >1% via targeted exome sequencing. Variant allele frequencies (VAFs) in primary and secondary recipients were plotted against the corresponding patient’s. Individual mice are represented by symbol shape and mutations are color coded. Linear regression, Pearson's correlations, and *p* values between patient and xenograft VAF are displayed. **i**, **j** Primary and secondary transplantation of a low-risk MDS-RS-SLD sample (Y007) comparing **i** overall engraftment in PB and BM and **j** multi-lineage representation in BM of primary and secondary NSG and MISTRG recipients. Individual mice are represented by symbols with means ± S.E.M.; symbols for corresponding 1° and 2° recipient mice are color coded; statistics represent Mann–Whitney test; n.s. not significant, *p < 0.05, **p < 0.01, ***p < 0.001, ****p < 0.0001. For patient information see Supplementary Table 1

We next determined whether MISTRG mice engraft human HSC via phenotypic^38,39^ and functional assays. CD34^+^ cells localize along the trabecular bone in MISTRG bone marrow (Fig. 5a and Supplementary Figure 6e). In addition to the overall increased engraftment (Fig. 2 and Supplementary Figure 2), MISTRG support phenotypic MDS HSCs as evident by flow cytometric analysis (Fig. 5b, c). The clonality of these MDS grafts was verified by targeted exome sequencing in representative mice (Supplementary Figure 6f, g and Supplementary Table 2).

Phenotypic identification of HSCs is insufficient to prove stem cell engraftment. Long-term engraftment (≥16 weeks) and functional assessment in the form of secondary engraftment are critical. Previous studies have shown successful secondary transplantation of AML^40,41^ and more recently of chronic and juvenile myelomonocytic leukemia  in NSG and NSG-SGM3 mice^42^ but no study has shown successful serial transplantation of MDS.

We therefore tested secondary transplantation of a higher and lower-risk MDS samples according to our standard protocol (Fig. 5d). Primary NSG and MISTRG recipient mice were maintained for ≥16 weeks. At the time of analysis, BM was enriched for human cells via bead depletion of murine CD45^+^ and Ter119^+^ cells (Supplementary Figure 6h, i) and transplanted intrahepatically into equal numbers of irradiated newborn mice of the respective strains. Secondary recipient mice were analyzed ≥12 weeks (unless otherwise noted) post 2° transplantation. For all samples tested, primary MISTRG recipient mice showed significantly higher overall engraftment levels (Figs. 2, 5e, i and Supplementary Figure 7a). These superior engraftment outcomes are also reflected in the significantly higher phenotypic stem cell frequency in 1° and 2° MISTRG compared to 1° and 2° NSG grafts (Fig. 5f and Supplementary Figure 7c, d, g) accompanied by myeloid predominant multi-lineage output (Fig. 5g, j and Supplementary Figure 7b, h, i) as well as erythro- and megakaryopoiesis (Supplementary Figure 4g–i). While engraftment levels were significantly lower for NSG mice, MDS clonality of primary and secondary grafts in MISTRG and NSG recipients was confirmed by targeted exome sequencing or cytogenetic analysis as indicated (Fig. 5h and Supplementary Figure 7e, j, k). The similar VAFs between MISTRG and NSG mice suggest that B cells, which predominate in NSG mice in MDS xenografts, are derived from the MDS clone.

Overall, these data show that MISTRG not only provide superior engraftment in primary recipients but also in serial transplantation, with propagation of clonal MDS stem cells that give rise to tri-lineage hematopoiesis with myeloid predominance and representation of hallmarks of dysplasia. This may at last fill the unmet need for MDS PDXs for the study of MDS disease mechanism and the development and testing of novel therapies.

### MISTRG MDS PDXs are ideally suited for pre-clinical modeling of targeted therapeutics

Targeted therapeutics provide novel opportunities for the treatment of MDS, but to date have failed to cure the disease. Recently, inhibitors of mutant IDH1/2 have entered clinical trials, and early data suggest that they result in blast differentiation and hematopoietic remissions, but fail to abrogate the mutant clone in the majority of patients^32,43,44^. While transgenic murine models can provide proof of principle data, patient-derived xenografts are critical to evaluate efficacy against complex clonal hematopoietic malignancies such as MDS and are likely to hasten development of valuable combination therapies.

We transplanted MISTRG mice with IDH2^R140Q^-mutant MDS-EB-2 CD34^+^ cells (Y021, Supplementary Table 1) and treated engrafted mice with either vehicle or enasidenib via oral gavage for 30 days. Mice were assigned to enasidenib or vehicle 16 weeks post transplantation based on equal engraftment levels as determined by BM aspiration (pre). Activity of enasidenib was verified in vitro via measurement of 2-hydroxy-glutarate (2-HG) levels in IDH2-wild-type (WT) and -mutant (MUT) expressing human erythroid leukemia (HEL) cell lines (Supplementary Figure 8a) and primary AML (Supplementary Figure 8b, Y029, Y031). Enasidenib efficiently suppressed 2-HG production and inhibited proliferation of IDH2^R140Q^- and IDH2^R172K^-mutant but not IDH2-wild-type AML cell lines and IDH2^R140Q^-mutant primary AML compared to vehicle and WT AML (Supplementary Figure 8c). Enasidenib treatment resulted in differentiation of IDH2 mutant myeloid blasts (Supplementary Figure 8d).

**Fig. 6:**
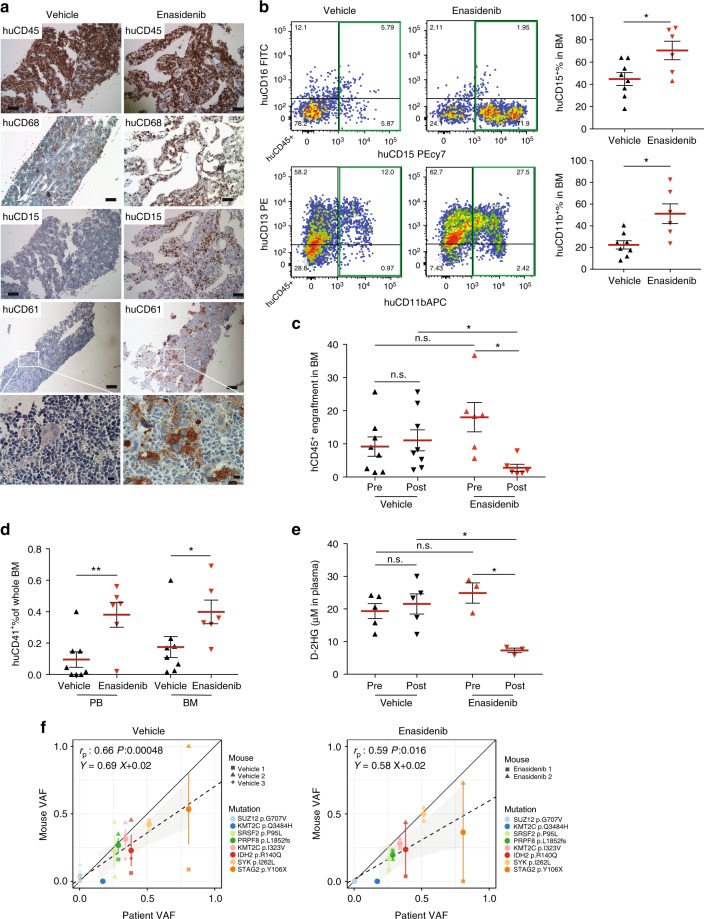
MISTRG replicate granulocytic and megakaryocytic differentiation in response to inhibition of mutant isocitrate dehydrogenase 2 (IDH2) in vivo. **a** In vivo treatment of mutant IDH2 R140Q in MDS-EB-2 (Y021)-engrafted MISTRG mice with the IDH2^MUT^ inhibitor enasidenib. Representative histologic images of vehicle-treated (*n* = 8, left) and enasidenib-treated (*n* = 6, right) mice engrafted with MDS-EB-2 (Y021). Immunohistochemistry (IHC) stains for huCD45, huCD68, huCD15, and huCD61 (scale bars 100 µm, original magnification 10×; high-power field 10 µm, original magnification 60×). **b** Representative fluorescence-activated cell sorting (FACS) plots showing myeloid maturation in response to enasidenib and quantitation of huCD15^+^ and huCD11b^+^ expression in vehicle- versus enasidenib-treated MISTRG mice. **c** Comparison of human engraftment in bone marrow (BM) from vehicle-treated (*n* = 8) and enasidenib-treated (*n* = 6) MISTRG mice. **d** Quantitation of huCD41^+^ expression in peripheral blood (PB) and BM from vehicle-treated (*n* = 8) and enasidenib-treated (*n* = 6) MISTRG mice. **e** Quantitation of D-2-HG in plasma of pre- and post-administration of vehicle or enasidenib. Individual mice are represented by symbols with mean ± S.E.M.; statistics represent Mann–Whitney test; n.s. not significant, *p < 0.05, **p < 0.01 for aggregate NSG vs. MISTRG. **f** Representation of variant allele frequencies (VAFs) of driver mutations in vehicle-treated (left) or enasidenib-treated (right) MISTRG (*y*-axis) plotted against the patient’s VAFs (*x*-axis). Individual mice represented by symbol shape, mutations color coded. Linear regressors, Pearson's correlations, and *p* values between patient and xenograft VAFs are displayed

Treatment with enasidenib, but not vehicle, resulted in myeloid differentiation in the IDH2^R140Q^ MDS-EB-2-engrafted MISTRG mice (Fig. 6a, hCD68 and hCD15 and Fig. 6b). Overall engraftment levels were significantly reduced in enasidenib-treated animals when compared to pre-treatment and vehicle-treated mice (Fig. 6c). Of note, enasidenib-treated mice also exhibited increased numbers of CD41^+^ platelets in PB and clustering megakaryocytes in BM but no difference in the erythroid lineage compared to vehicle-treated mice. (Fig. 6a (huCD61), Fig. 6d and Supplementary Figure 8e, f, g). Plasma 2-HG levels in vivo, elevated pre-treatment and in vehicle-treated MISTRG mice, were significantly suppressed after administration of enasidenib (Fig. 6e). Variant allele frequencies of mutations identified in the patient were represented in all MISTRG mice and not significantly altered by enasidenib treatment (Fig. 6f, Supplementary Table 2).

MISTRG PDX represent the first MDS pre-clinical model that allows to test not only for cytotoxic but also for differentiating effects of targeted therapeutics and capturing multi-faceted responses relevant to clinical success.

## Discussion

MDS is a disease of the hematopoietic stem cell and studies of MDS have been hampered by the inability to expand HSCs in general and MDS stem cells in particular. There is an unmet need for an in vivo pre-clinical model to accelerate development of novel treatments for a disease where allogeneic stem cell transplantation currently represents the only cure. Mouse models only partially recapitulate the genetic and epigenetic complexity of patients’ MDS. Prior xenotransplantation studies have allowed identification of the MDS HSC^38,39^, but have been hampered by preferential engraftment of the remnant normal hematopoiesis^10^, transient engraftment^9^, and low efficiency with low engraftment levels of only a subset of samples^38,39,45^. Cytokine humanization via transgenic expression in the NSG-SGM3 mice, while advantageous in AML^12^ and other myeloid malignancies^42^, impairs stem cell function^11,14^ and provides limited advantages over NSG mice for MDS engraftment^15,45^. Co-injection of human MSC may provide transient support to MDS HSC^16,45^ and generation of a human niche via growth of human MSC-derived ossicles may afford improved engraftment of HSCs^17,46^ and difficult-to-engraft leukemias^46^, but applicability in pre-clinical models at a large scale is likely limited due to technical complexity.

MISTRG mice were engineered to express key non-crossreactive human cytokines from the endogenous murine loci in place of their murine counterparts, thereby providing temporally and spatially physiologic expression of human cytokines. In addition, lack of murine cytokines reasonably provides additional benefit to human hematopoiesis by rendering the stem and progenitor niches in the BM less hospitable to murine HSPC. This is likely to be particularly critical to adult HSCs that have markedly lower proliferative and self-renewal capacity than their fetal liver and umbilical cord blood  counterparts (reviewed in ref. ^47^). In addition, MDS stem cells frequently fail to give rise to colony-forming units in vitro, a manifestation of their defective proliferative and differentiation capacity. Research material from human BMs is limited and worse in aging marrows that are characterized by progressively lower cellularity.

Here, we present for the first time a highly efficient and versatile xenotransplantation model for MDS. We show that MISTRG mice can be engrafted with as few as ~1.5 × 10^5^ MDS BM-derived HSPCs. Higher engraftment levels clearly improve MISTRG utility as a model. Over 80% of MISTRG mice engraft when a threshold of 1% BM huCD45^+^ cells is set. More remarkably, over 50% of MISTRG mice, compared to fewer than 10% of NSG mice, engraft above a threshold of 10% BM huCD45^+^ cells. In addition, MISTRG mice persistently show improved myeloid representation and differentiation, as evident by both flow cytometry and histologic evaluation.

Study of adult erythropoiesis and megakaryopoiesis in in vivo models has been elusive to date. MDS is characterized by cytopenias in the peripheral blood, left-shifted myeloid maturation, and erythroid and megakaryocytic dysplasia^3^. Very little is known about the causes of the phenotypic heterogeneity in MDS and genotype–phenotype studies would greatly advance our mechanistic understanding of this complex entity. To date, immunodeficient mouse models have supported erythro- and megakaryopoiesis solely from fetal liver- and cord blood-derived HSPCs^48,49^, further enhanced by mutation of the murine ckit receptor conferring impaired function to murine stem cells^50–52^ and likely murine erythropoietic progenitors^53^. None of these models have supported erythro- and megakaryopoiesis from adult HSPCs. We propose that while cytokine humanization directly enhances overall engraftment and myeloid maturation, lack of the corresponding murine cytokines impairs murine hematopoiesis, thereby synergistically promoting human HSPC competitiveness in the mouse niche. As a result, we here show for the first-time development of both erythropoiesis and megakaryopoiesis from healthy adult and MDS BM in a murine host. This may be further aided by introduction of the murine c-kit mutation into MISTRG^51,54^. Intriguingly, MDS-engrafted MISTRG mice replicate the patient’s erythroid and megakaryocytic dysplasia, with development of ring sideroblasts and dysplastic megakaryocytes with reticulin fibrosis, making MISTRG MDS PDXs uniquely suited to assess MDS-associated abnormalities in all three myeloid lineages.

We have previously reported that MISTRG life span is limited in fetal liver engraftment due to destruction of murine RBC and platelets by human macrophages^18^. Interestingly, MDS-engrafted MISTRG lack significant development of cytopenias and their life span is similar to that of engrafted NSG mice. One possible explanation is the lower engraftment compared to fetal liver HSPC, yet mice engrafted with normal adult CD34^+^ cells with similar engraftment levels to MDS-engrafted mice show evidence of hemophagocytosis by human macrophages ^23^. In contrast to normal CD34^+^ engrafted MISTRG, MDS PDX lack human-derived tissue macrophages in spleen and liver, confirming a functional defect of MDS-derived mature myeloid cells that likely also lack in vivo hemophagocytic activity. Interestingly, MDS blasts, unlike in AML, do not infiltrate non-hematopoietic tissues, functionally distinguishing MDS also from AML in MISTRG PDX.

Cytokine humanization does not alter the lack of mature human red blood cells (RBCs) and the low human platelet percentage in the peripheral blood as also shown previously^18,23^. Administration of human erythropoietin has shown no benefit in this regard^54^ as the defect lies in RBC and platelet destruction by the murine innate immune system. Thus, modulation of the murine innate immune system will be necessary to promote mature human cell persistence in peripheral blood, transiently achieved by administration of liposome-encapsulated clodronate^49,55^.

MDS is a clonal hematopoietic stem cell disorder and reliable engraftment of the malignant HSC is essential for high-quality pre-clinical studies of disease biology and response to therapeutics. While phenotypic evidence of HSC can suggest their presence, functional assays are critical. Serial transplantation represents the gold-standard functional hematopoietic stem cell assay. We here show successful serial transplantation of MDS into MISTRG secondary recipients with faithful representation of the clonal composition and lineage representation of the parental patients’ BMs in primary and secondary recipients. Importantly, MISTRG mice allow the expansion of xenografts from one primary into several secondary recipients, essential for pre-clinical modeling and therapeutic testing.

We interrogated the utility of the MISTRG MDS PDX model in the testing of targeted therapeutics, specifically inhibition of mutant IDH2. Early clinical studies have shown that enasidenib, an oral inhibitor of mutant IDH2, results in differentiation of mutant myeloblasts without abrogation of the mutant clone in the majority of patients. We here show for the first time, in an in vivo MDS PDX model, differentiation towards dysplastic megakaryocytes and myeloid maturation with preservation of the clonal composition of the graft. The MISTRG MDS PDX model is ideally suited for the systematic study of targeted therapeutics alone and in combination with other agents. Concurrent targeted exome sequencing may allow predicting ideal combination regimens for individual patients.

In summary, we here present a highly efficient, faithful MDS PDX model, ideally suited for the study of MDS biology, the development of novel treatment approaches, and adaptation of patient-specific regimens in the era of precision medicine.

## Methods

### Human progenitor cell isolation

Peripheral blood, BM, and umbilical cord blood were obtained with donor’s written consent. All human studies were approved by the Yale University Human Investigation Committee and by the West Haven Veterans Affairs Human Investigation Committee.

Human BM, cord blood, and peripheral blood samples were ficolled (GE Healthcare, Munich, Germany) and mononuclear cells cryopreserved within 24 h after collection in fetal bovine serum/10% dimethyl sulfoxide. Samples were CD34 enriched with the CD34-Microbead-Kit or T cell depleted via negative selection with the CD3-Microbead-Kit (Miltenyi-Biotech, Bergisch-Gladbach, Germany). CD34-enriched or CD3-depleted HSPCs were incubated with a murine anti-human CD3 antibody (clone Okt3, BioXCell, NH, USA) at 5 µg/100 µl for 10 min at room temperature prior to injection into mice.

### Generation and analysis of MISTRG PDXs

All animal experiments were approved by the Institutional Animal Care and Use Committee of Yale University. Mouse breeding and xenografting: MIS^h/h^TRG mice with homozygous knockin replacement of the endogenous mouse *Csf1, Il3, Csf2, Tpo*, and *Sirpa* with their human counterparts were bred to MITRG mice to generate human cytokine homozygous and *hSIRPA* heterozygous mice^18,19^. MISTRG mice have been deposited at Jackson laboratory. Mice will be available via MTA and requests should be sent to mistrg@yale.edu. NSG mice were obtained from Jackson laboratory. MIS^h/m^TRG (labeled MISTRG throughout the study) and NSG mice were maintained on continuous treatment with enrofloxacin in the drinking water (0.27 mg/mL, Baytril, Bayer Healthcare). Newborn MISTRG or NSG mice (1 to 3 days of age) were sublethally irradiated (X-ray irradiation with X-RAD 320 irradiator; MISTRG 2 × 150 cGy 4 h apart, NSG 1 × 100 cGy). Equal numbers of split-donor MDS BM CD34-selected or CD3-depleted (as indicated) were injected intrahepatically in a volume of 20 µL into with a 22-gauge Hamilton needle (Hamilton, Reno, NV). Mice were analyzed at least 12 weeks post transplantation and only sooner if moribund. For secondary transplantation, human cells were isolated from primary recipient BMs and depleted of murine cells via negative selection of murine CD45^+^ and Ter119^+^ cells by magnetic labeling with biotin-anti-muCD45 (clone 30-F11, Biolegend, San Diego, CA) and muTer119 (clone TER-119, Biolegend) and BD IMag Streptavidin Particles (BD Biosciences, San Jose, CA).

#### Flow cytometric analysis

Engraftment of human CD45^+^ cells and their stem cell, progenitor, and mature myeloid, lymphoid, and erythroid or megakaryocytic subsets were determined by flow cytometry using antibody panels detailed in Supplementary Table 3. In brief, cells were isolated from engrafted mice, blocked with human/murine Fc block, and stained with indicated combinations of antibodies. Data were acquired with FACSDiva on a LSR Fortessa (BD Biosciences) equipped with 5 lasers and analyzed with FlowJo V10 software.

#### Histologic analysis

Tissues were fixed in Bouin’s Fixative solution (RICCA Chemical Company, TX, USA) and embedded in paraffin. Femurs were decalcified with Formic Acid Bone Decalcifier (Decal Chemical, NY, USA). Paraffin blocks were sectioned at 4 μm and stained with hematoxylin and eosin (H&E) or antigen-specific antibodies routinely used in the Yale Clinical Pathology and Yale Pathology Tissue Services (Supplementary Table 4). Images were acquired using Nikon Eclipse 80i microscope. Bone marrow aspirate smears were stained with Prussian Blue Iron stain per standard protocol.

All animal experimentations were performed in compliance with Yale Institutional Animal Care and Use Committee protocols.

### Targeted exome capture and sequencing and analysis

DNA was digested using the QIAamp DNeasy blood and tissue DNA extraction kits (Qiagen), according to the manufacturer’s recommendations. Purity and concentration of the extracted DNA was measured using NanoDrop 1000 spectrophotometer (Thermo Scientific) and Quant-iT PicoGreen dsDNA Assay Kit (Life Technologies, Carlsbad, CA) for all samples.

A library of coding exons and intron–exon boundaries of 142 genes (see Supplemental Table 6) known to carry mutations in myeloid malignancies and cancers was prepared using the HaloPlex target enrichment kits and HaloPlex HS Target Enrichment System (Agilent Technologies, Santa Clara, CA) according to the manufacturer’s instructions. In brief, approximately 200 ng of DNA was fragmented using restriction enzymes proprietary to the kit. For mixed human/mouse samples isolated from MISTRG mice total DNA (human/mouse) input was calculated with the endpoint of 200 ng human DNA input based on engraftment percentage of huCD45^+^ muCD45^−^ cells determined by flow cytometry. Probes with sequence indexes were hybridized to the targeted DNA fragments. Each probe is designed to hybridize to both ends of a targeted DNA restriction fragment resulting in their circularization. The biotinylated probe-DNA fragment hybrids were retrieved with magnetic streptavidin beads. Small fragments of <150 bp and unligated probes were removed from the mix by AMPure purification (Agencourt Bioscience, Beverly, MA). Circular molecules were ligated and enriched DNA fragments were amplified with universal primers. Quality of the libraries was verified using the Tape Station 4200 (Agilent) and input DNA estimated using a library quantification kit (Kapa Biosystems, Wilmington, MA, USA). For samples Y013, Y014, Y016, Y019, Y021, Y022, Y028, Y029, and their engrafted NSG and MISTRG mice, a second-generation enrichment kit was used with Agilent’s improved high-sensitivity technology with addition of molecular barcodes to each probe. Sequencing was performed on Illumina HiSeq 2000 using 74 base pairs paired-end reads, HiSeq 4000 using 100 base pairs paired-end reads, or MiSeq using 250 base pairs paired-end reads. Reads were filtered by Illumina CASAVA 1.8.2 software, and trimmed at the 3’ end using FASTX v0.0.13. To remove potential mouse contamination, each read pair was aligned to a concatenated genome of human (GRCh37) and mouse (mm10) reference genome by Burrows-Wheeler Aligner v0.7.5a. Only read pairs that were specifically aligned to human reference genome were extracted for the downstream analysis. Local realignment was performed around putative and known insertion/deletion (INDEL) sites using RealignerTargetCreator (Genome Analysis Toolkit: GATK v3.1.1) and applied base quality recalibration using GATK. MuTect v.1.1.4 and Strelka v.1.0.14 were applied to call somatic single-nucleotide variants and indels, respectively. Whole-exome sequencing data from 10 external normal blood samples were pooled to serve as reference normal cohort for somatic variant calling by MuTect and Strelka. In each sample, low confidence somatic calls were removed by applying the following filters: (i) variants with total coverage <50, (ii) with a ratio of mutant allele frequency (MAF) in tumor versus normal <5, and (iii) variant base quality <20. Variants that were considered likely to be germline because they were listed in any of the following datasets, dbSNP, ESP6500, 1000Genome, or Exac01, or had MAF <0.02 in the tumor samples were excluded from further analysis. Recurrent (*N* > 5 cases) annotated variants in COSMIC v64 and Clinvar (http://www.ncbi.nlm.nih.gov/clinvar/) were white-listed. At last, only non-synonymous variants were kept. To extract the allele frequency of the variants, all non-synonymous somatic mutations from all the samples associated with certain patients were aggregated. The allele frequency of each variant was assessed using Samtools mpileup.

### Enasidenib treatment

Enasidenib was purchased from Shanghai ZaiQi Bio-Tech, China, and LeadGen Labs, USA, and quality verified by the Yale Center for Molecular Discovery. Cell proliferation and suppression of 2-HG production was verified in vitro by treating dox-induced HEL-IDH2 WT/R172K/R140Q cells and primary human leukemia cells. Primary IDH2 WT and R140Q leukemia MNC were cultured in StemSpan (Stem Cell Technologies, Vancouver, Canada) supplemented with 1% Penicillin/Streptomycin and recombinant human cytokines FLT-3 (50 ng/mL), SCF (50 ng/mL), THPO (100 ng/mL), IL-3 (10 ng/mL), and IL-6 (25 ng/mL). All cytokines were purchased from NeoBioSci, MA, USA.

Enasidenib dosing was optimized and added to cell cultures (20 nM) every other day. For in vivo treatment, enasidenib was dissolved in 0.5% methylcellulose and 0.2% Tween-80 in phosphate-buffered saline at a concentration of 4 mg/mL and administered via oral gavage twice daily at 40 mg/kg. 2-HG was measured in cell culture supernatants and in plasma of IDH2^WT^ and IDH2^R140Q^ leukemia-engrafted MISTRG before and after treatment with enasidenib or vehicle. 2-HG was measured in triplicate with the D-2-hydroxyglutarate (D2HG) Assay kit (Biovision, CA, USA) according to the manufacturer’s protocol.

### Statistical analysis

Data were analyzed using Prism 7 (GraphPad Software, La Jolla, CA) using Fisher’s exact test, unpaired *t*-test, unpaired Mann–Whitney *U* test, one-way analysis of variance (ANOVA) Kruskal–Wallis test with Dunn’s Multiple Comparison Test, or two-way ANOVA with Sidak’s Multiple Comparison Test as indicated. The *p* value was considered significant at values less than 0.05, n.s. not significant, statistically significant (*p < 0.05, **p < 0.01, ***p < 0.001, ****p < 0.0001). Linear regression analyses were performed with R. Pearson's correlation *p* values were determined with the cor.test () function implemented in R.

### Code availability

All data analysis codes are available upon request.

### Reporting summary

Further information on experimental design is available in the Nature Research Reporting Summary linked to this article.

## Supplementary information

Supplementary Information

Reporting Summary

## Data Availability

Sequencing data have been deposited into dbGaP under the Study ID 32505. Additional data could not be uploaded due to ethical permissions and is available upon request.
