# Supplementary material for: A highly efficient and faithful MDS patient-derived xenotransplantation model for pre-clinical studies
Source: Nat Commun. 2019 Jan 21;10:366. doi: 10.1038/s41467-018-08166-x (PMC6341122; doi:10.1038/s41467-018-08166-x)
Supplement: Supplementary file 1 — Supplementary Information [file 41467_2018_8166_MOESM1_ESM.pdf]

A Highly Efficient and Faithful MDS Patient-Derived Xenotransplantation Model for Pre-Clinical Studies

Supplementary Materials

Song et al.

Supplementary Methods

Cloning and Isogenic Cell Line Construction

To generate isogenic wildtype and mutant expressing cell lines the lentiviral plasmids pSLIK-IDH2-FLAG and pSLIK-IDH2-R172K-FLAG (a gift from Christian Metallo, Addgene plasmid ## 66806, 6680, [48]) were used to transduce human erythroid leukemia cell (HEL) cell lines. To generate IDH2 R140Q mutant plasmid IDH2-WT-FLAG was amplified and cloned into pGEM®-T Easy Vector Systems (A1360, Promega, Madison, WI). IDH2 R140Q site-directed mutagenesis was preformed using the QuikChange II Site-Directed Mutagenesis Kit (Agilent Technologies) with primers provided in **Supplementary Table 5** and cloned into the pEN_TTmcs entry vector for recombination into the pSLIK-hygro lentiviral vector (kind gifts from Iain Fraser, Addgene plasmid # 25755 and # 25737, respectively, [49]). Viral particles were produced by cotransfection of 293FT cells (Life Technologies) with psPAX2 (a gift from Didier Trono (Addgene plasmid # 12260)) and pCMV-VSVG (a gift from Tannishtha Reya (Addgene plasmid # 14888)) using lipofectamine transfection reagent (Life Technologies). HEL cells were grown in RPMI/10% FBS/1% P/S/G and transduced at a multiplicity of infection (MOI) of 1, Hygromycin selected to generate HEL/ IDH2 WT, R172K and R140Q expressing cell lines. Doxycycline-inducible expression (1mg/ml, Sigma-Aldrich, St Louis, MO) was verified by sanger sequencing and western blotting with anti-Flag antibody (Clone M2, Sigma-Aldrich).

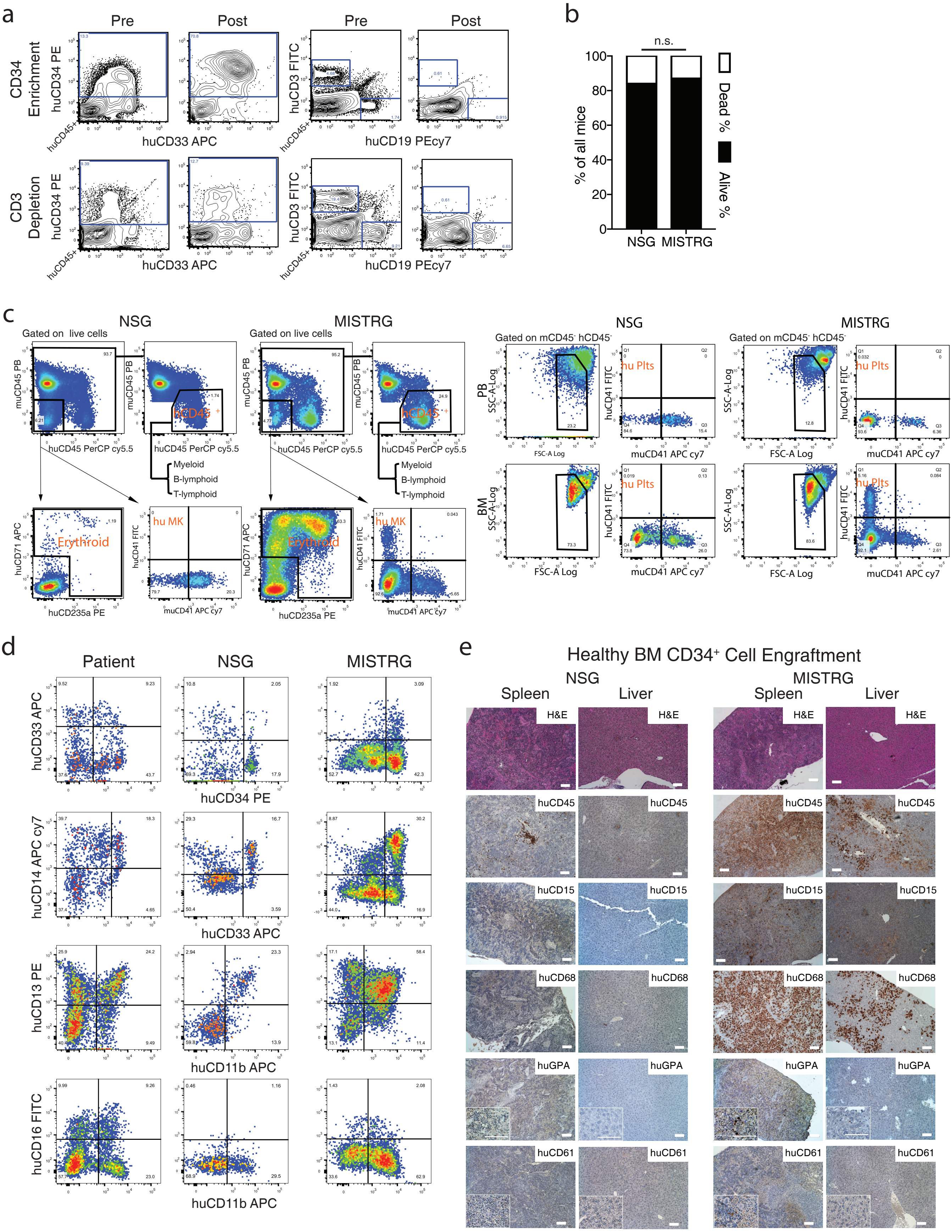
a
Pre
Post
Pre
Post
CD34 Enrichment
huCD34 PE
huCD33 APC
huCD3 FITC
huCD19 PEcy7
huCD45+
CD3 Depletion
b
n.s.
% of all mice
Dead %
Alive %
NSG
MISTRG
c
NSG
MISTRG
Gated on live cells
muCD45 PB
huCD45 PerCP cy5.5
hCD45+
Myeloid
B-lymphoid
T-lymphoid
huCD71 APC
huCD235a PE
Erythroid
huCD41 FITC
muCD41 APC cy7
hu MK
Gated on mCD45- hCD45-
PB
BM
SSC-A Log
FSC-A Log
hu Plts
d
Patient
NSG
MISTRG
huCD33 APC
huCD34 PE
huCD14 APC cy7
huCD13 PE
huCD11b APC
huCD16 FITC
e
Healthy BM CD34+ Cell Engraftment
NSG
MISTRG
Spleen
Liver
H&E
huCD45
huCD15
huCD68
huGPA
huCD61

Supplementary Figure 1: CD34-enrichment and T-cell depletion of xenografts, flow-cytometric analysis of myeloid lineage differentiation, and liver and spleen histologies. Related to Fig 1.

a Flow cytometric verification of $CD34^+$ enrichment (top row, representative example of patient samples, n=22) and CD3 depletion (bottom row, representative example of patient samples n=5) via magnetic bead separation of primary patient samples. **b** Survival in NSG (111/126) and MISTRG (154/186) mice engrafted with split-donor samples (Student's t-test n.s.). **c** Flow analysis gating strategies for engrafted NSG or MISTRG mice. Single cell suspensions were stained as in methods and plots gated on live single cells. For analysis of human leukocyte engraftment cells were gated on human vs murine CD45 and huCD45 engraftment was calculated as % of all $CD45^+$ cells, presented on Fig.1b, c; Fig.2; Fig.4c; Fig.5e, I; Fig.6c. For analysis of the erythroid and megakaryocytic lineages the human and murine CD45 negative fraction was further analyzed for $huCD71^{bright/+}$, $huCD235^+$, $huCD41^+$ and muCD41, Erythroid lineage engraftment was calculated as $huCD71^{bright/+}$ and $huCD235^+$ % of total BM cells and megakaryocytic lineages engraftment was calculated as $huCD41^+$ and $muCD41^-$ % of total BM cells, presented on Fig.1f, g; Fig.3; Fig.6d. **d** Representative FACS dot plots comparing myeloid maturation of primary BM sample (top) and human xenografts in NSG and MISTRG mice xenografted with healthy BM (Y003) analyzed 13 weeks post-transplantation. MISTRG mice give rise to SSC^{high} myeloid cells with expression of mature myeloid differentiation markers. **e** Spleen and Liver histology of representative NSG and MISTRG mice engrafted with healthy adult $CD34^+$ (Y003 of n=3). H&E and IHC stains for huCD45, huCD15, huCD68, huGPA (huCD235), and huCD61 in NSG (top) and MISTRG BM (bottom rows) (scale bars 100µm, original magnification 10x).

Pre, post – before and after magnetic bead separation, CTRL control

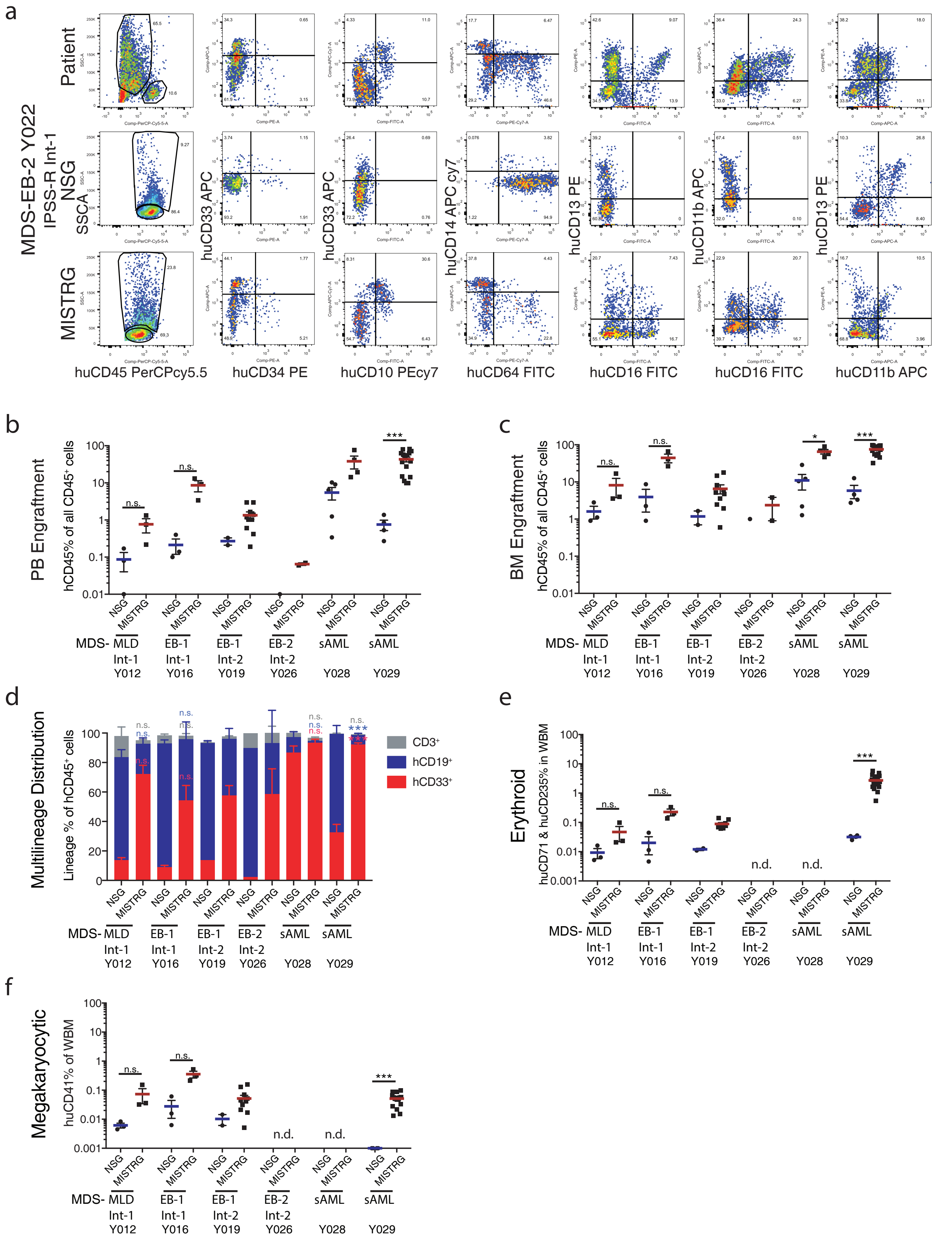
a
MDS-EB-2 Y022
IPSS-R Int-1
Patient
NSG
MISTRG
SSCA
huCD33 APC
huCD14 APC cy7
huCD13 PE
huCD11b APC
huCD45 PerCPcy5.5
huCD34 PE
huCD10 PEcy7
huCD64 FITC
huCD16 FITC
huCD11b APC
b
PB Engraftment
hCD45% of all CD45+ cells
c
BM Engraftment
NSG
MISTRG
MDS- MLD Int-1 Y012
EB-1 Int-1 Y016
EB-1 Int-2 Y019
EB-2 Int-2 Y026
sAML Y028
sAML Y029
n.s.
***
*
d
Multilineage Distribution
Lineage % of hCD45+ cells
CD3+
hCD19+
hCD33+
e
Erythroid
huCD71 & huCD235% in WBM
n.d.
f
Megakaryocytic
huCD41% of WBM

Supplementary Figure 2: MDS myeloid lineage differentiation engraftment, engraftment statistics, and engraftment of CD3-depleted MDS xenografts.
Related to Fig 2.
a FACS dot plots comparing parental MDS-EB-2 (Y022) patient with representative NSG and MISTRG mice engrafted > 1%. Myeloid maturation is enhanced in MISTRG mice. **b-f** Engraftment of CD3-depleted patient derived bone marrow samples. Comparison of human engraftment in **b** PB and **c** BM, and **d** multilineage **e** erythroid, and **f** megakaryocytic representation in NSG and MISTRG xenografts. Individual mice are represented by symbols with mean ± S.E.M. For detailed patient information see Supplementary Table 1. Mann Whitney test; n.s. not significant, ***$p<0.001$ for aggregate NSG vs. MISTRG.

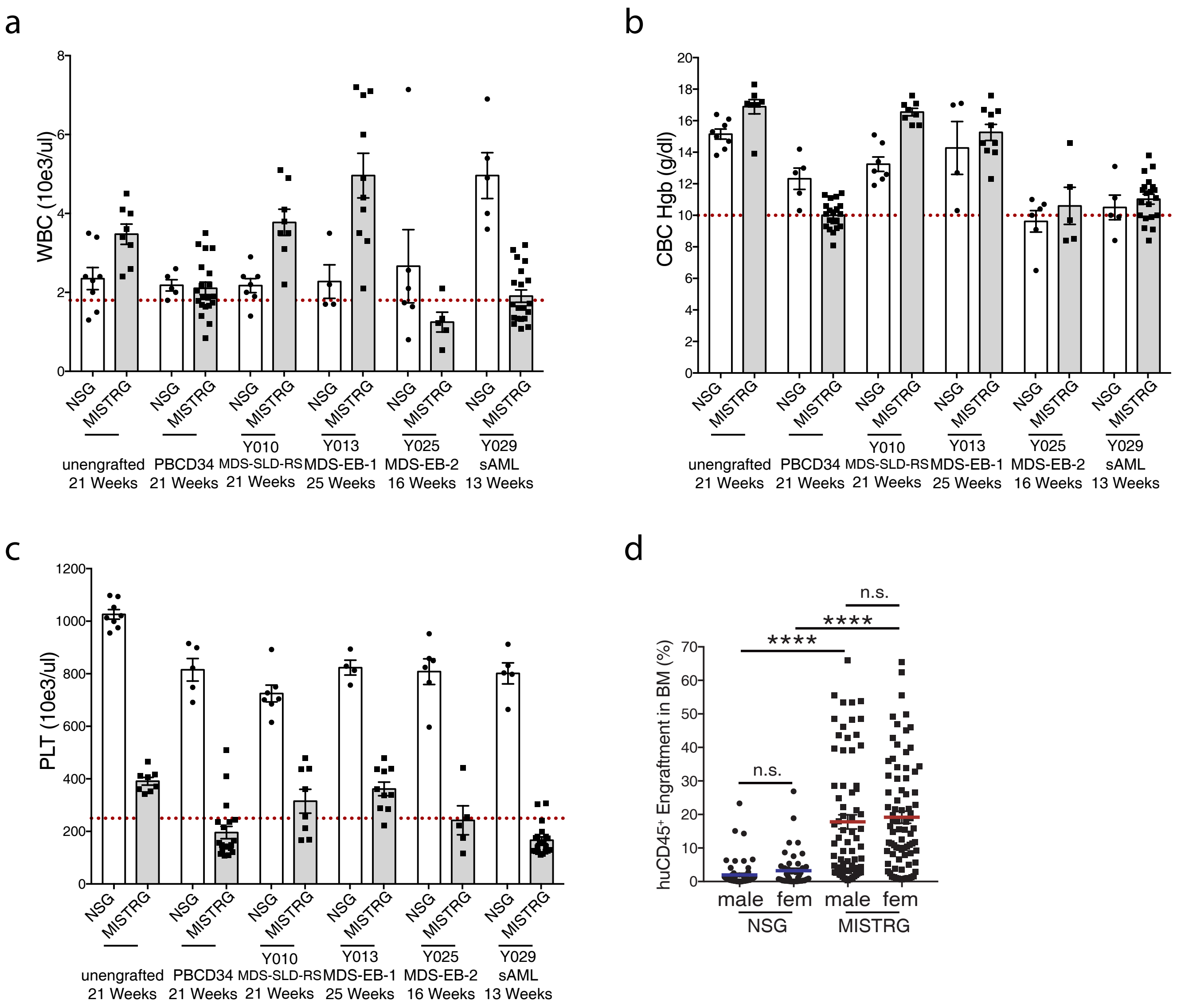

Supplementary Figure 3: Complete blood counts and gender statistics. Related to Fig 2. a-c Complete counts showing **a** white blood cell counts (WBC), **b** Hemoglobin (Hgb), **c** and platelet counts (PLT) in unengrafted and engrafted (with normal CD34+ cells, MDS, and sAML) NSG and MISTRG recipient mice. **d** Analysis of mouse recipient gender on engraftment levels. Bar graphs show mean ± S.E.M. engraftment levels (BM huCD45^{+} %) in split-donor xenografted NSG (male n=64, female n=47) and MISTRG (male n=73, female n=81) mice. MISTRG show overall higher engraftment without significant difference between female and male recipient mice. Statistical parameters represent nonparametric One way ANOVA, Dunn's multiple pairs comparison, NSG male vs. female n.s., NSG male vs. MISTRG male ****p<0.0001, NSG female vs. MISTRG female ****p<0.0001, MISTRG male vs. female n.s.).

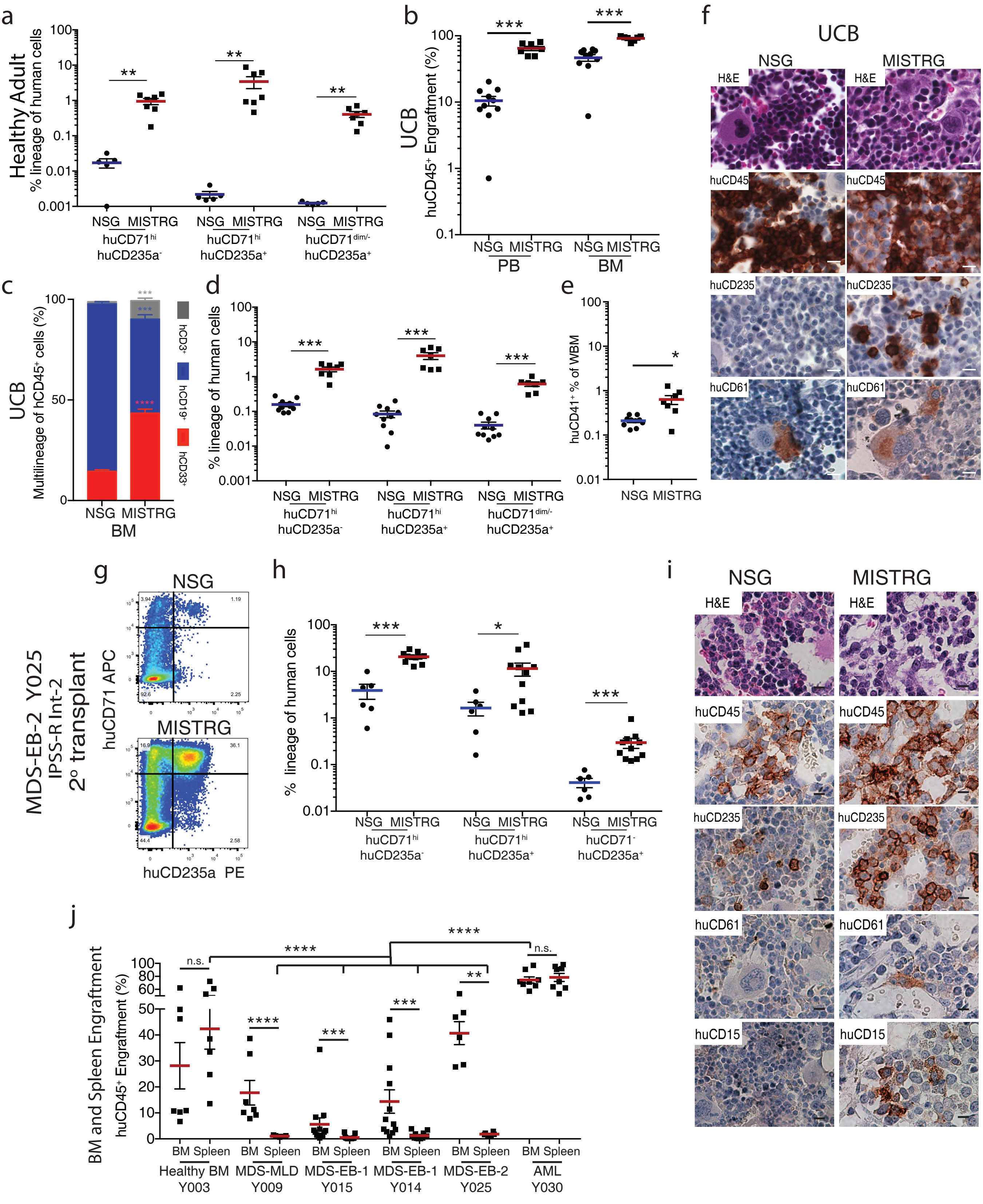
a
Healthy Adult
% lineage of human cells
NSG MISTRG
huCD71hi huCD235a-
huCD71hi huCD235a+
huCD71dim/- huCD235a+
b
UCB
huCD45+ Engraftment (%)
NSG MISTRG
PB
BM
c
UCB
Multilineage of hCD45+ cells (%)
hCD3+
hCD19+
hCD33+
BM
d
% lineage of human cells
e
huCD41+ % of WBM
f
UCB
NSG
MISTRG
H&E
huCD45
huCD235
huCD61
g
MDS-EB-2 Y025
IPSS-R Int-2
2° transplant
huCD71 APC
huCD235a PE
h
% lineage of human cells
huCD71- huCD235a+
i
huCD15
j
BM and Spleen Engraftment
huCD45+ Engraftment (%)
n.s.
BM Spleen
Healthy BM Y003
MDS-MLD Y009
MDS-EB-1 Y015
MDS-EB-1 Y014
MDS-EB-2 Y025
AML Y030

Supplementary Figure 4: Erythroid engraftment and preserved erythroid lineage output in serial transplantation. Related to Fig 3.

a-f MISTRG support healthy adult and human umbilical cord blood (UCB) -derived erythropoiesis. **a** Representative example of healthy adult BM $CD34^{+}$-derived erythropoiesis in NSG and MISTRG recipient mice. **b** Comparison of hUCB engraftment in PB and BM, **c** multilineage engraftment in BM in NSG (n=10) versus MISTRG (n=7); **d** erythroid lineage representation; and **f** Representative BM histology slides from hUCB engrafted NSG vs. MISTRG recipient mice. (Scale bars 10µm, original magnification 60X). **e-g** Representative FACS plots **e**, quantitation **f**, and histology **g** of MDS-EB2 (Y025) engrafted secondary NSG (n=6) and MISTRG (n=11) recipient mice with **h** quantification of erythroid lineage engraftment in secondary recipient mice and **i** representative histology images (Scale bars 10µm, original magnification 60X). Individual mice are represented by symbols with mean ± S.E.M. Mann Whitney test; *p<0.05, **p<0.01, ***p<0.001 for aggregate NSG vs. MISTRG. For detailed patient information see Supplementary Table 1. **j** Comparison of human engraftment in BM and spleen of MISTRG engrafted with healthy adult BM-, MDS-, or AML-derived patient sample. Mann Whitney test; n.s. not significant, **p<0.01, ***p<0.001, ****p<0.0001 for aggregate BM vs Spleen.

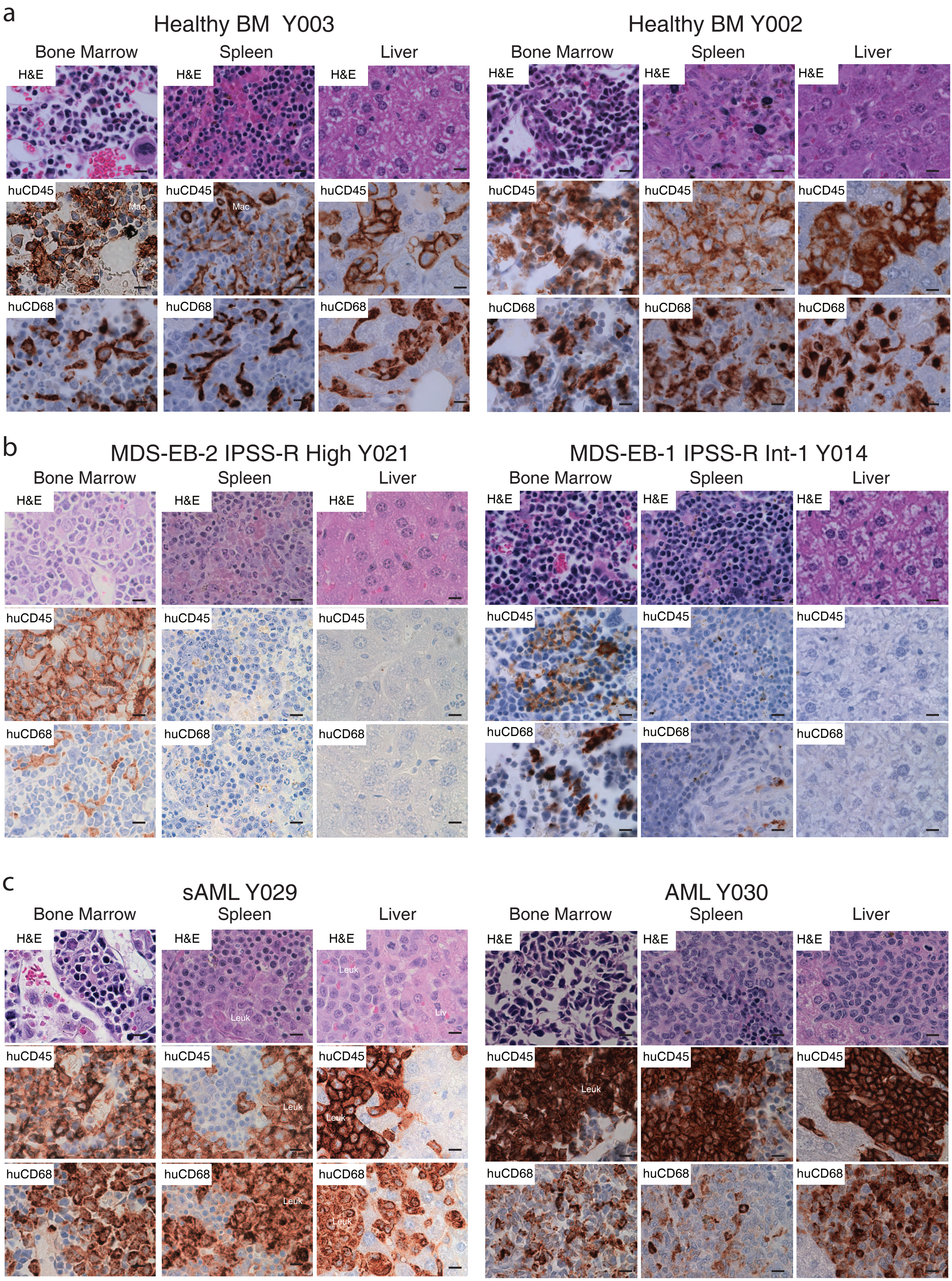

a
Healthy BM Y003
Healthy BM Y002
Bone Marrow
Spleen
Liver
H&E
huCD45
huCD68
Mac
b
MDS-EB-2 IPSS-R High Y021
MDS-EB-1 IPSS-R Int-1 Y014
c
sAML Y029
AML Y030
Leuk
Liv

Supplementary Figure 5: MISTRG mice replicate abnormal function of MDS myeloid cells and tissue infiltrative behavior of myeloid leukemia blasts, absent in MDS blasts. Related to Fig 4.

Histologic assessment of human myeloid cell tissue tropism in MISTRG mice engrafted with BM-derived $CD34^+$ cells from **a** healthy adult (Y002, Y003)**, b** MDS-EB-1 (Y014) and MDS-EB-2 (Y021), and **c** secondary sAML (Y029) and primary AML (Y030)**.** H&E as well as IHC stain for huCD45 and huCD68 cells in engrafted MISTRG BM (left), spleen (middle) and liver (right) (scale bars for high-power field: 10μm, original magnification 60X). Normal BM derived myeloid cells home to spleen and non-hematopoietic tissue (liver), while MDS BM derived myeloid cells fail to do so. Leukemic blasts infiltrate spleen and non-hematopoietic tissues ablating tissue architecture.

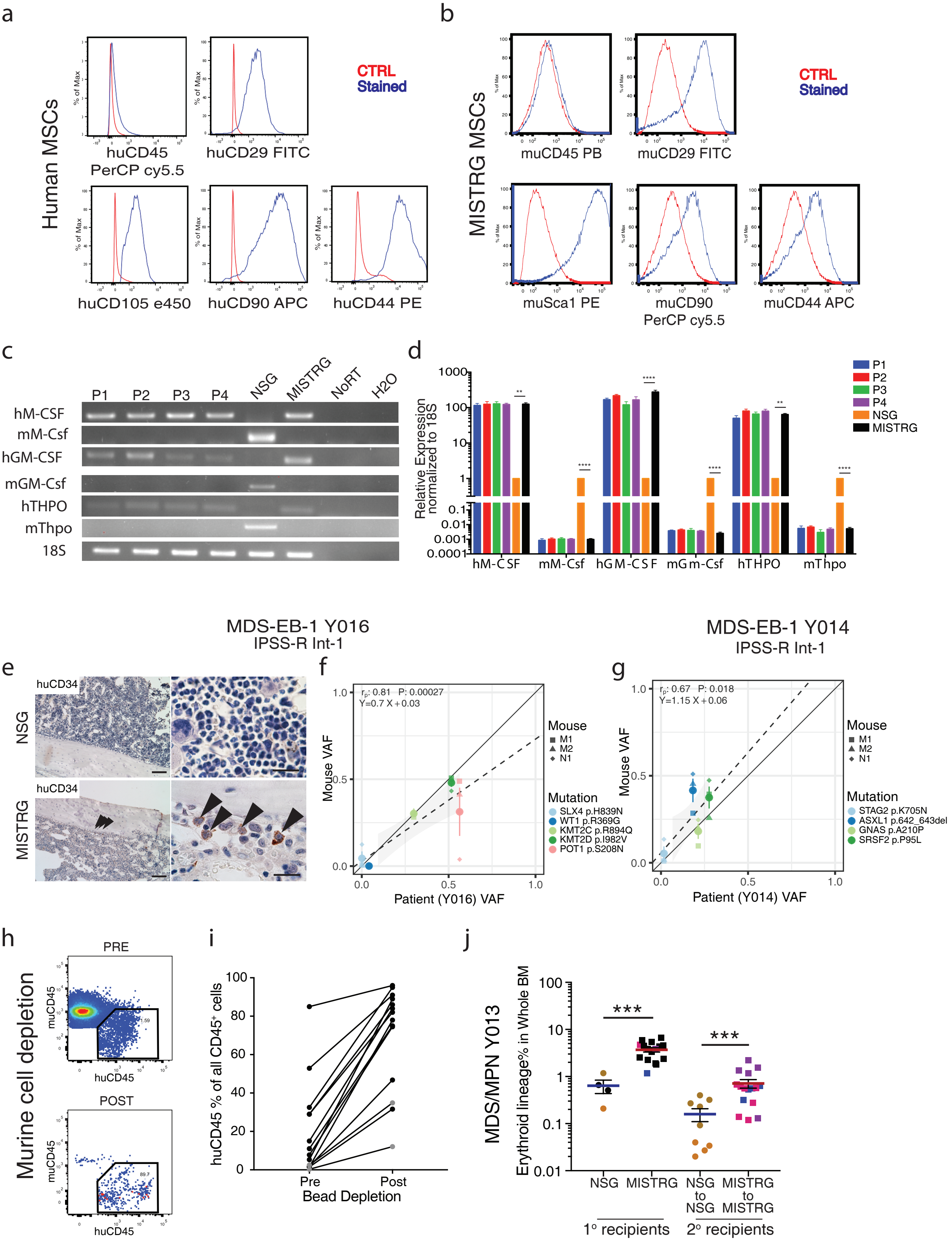
a
Human MSCs
huCD45 PerCP cy5.5
huCD29 FITC
huCD105 e450
huCD90 APC
huCD44 PE
CTRL
Stained
b
MISTRG MSCs
muCD45 PB
muCD29 FITC
muSca1 PE
muCD90 PerCP cy5.5
muCD44 APC
CTRL
Stained
c
P1 P2 P3 P4 NSG MISTRG NoRT H2O
hM-CSF
mM-Csf
hGM-CSF
mGM-Csf
hTHPO
mThpo
18S
d
Relative Expression normalized to 18S
hM-CSF mM-Csf hGM-CSF mGm-Csf hTHPO mThpo
P1 P2 P3 P4 NSG MISTRG
MDS-EB-1 Y016
IPSS-R Int-1
MDS-EB-1 Y014
IPSS-R Int-1
e
huCD34
NSG
MISTRG
f
rp: 0.81 P: 0.00027
Y=0.7 X+0.03
Mouse VAF
Patient (Y016) VAF
Mouse
M1
M2
N1
Mutation
SLX4 p.H839N
WT1 p.R369G
KMT2C p.R894Q
KMT2D p.I982V
POT1 p.S208N
g
rp: 0.67 P: 0.018
Y=1.15 X+0.06
Mouse VAF
Patient (Y014) VAF
Mouse
M1
M2
N1
Mutation
STAG2 p.K705N
ASXL1 p.642_643del
GNAS p.A210P
SRSF2 p.P95L
h
Murine cell depletion
PRE
POST
muCD45
huCD45
i
huCD45 % of all CD45+ cells
Pre
Post
Bead Depletion
j
MDS/MPN Y013
Erythroid lineage% in Whole BM
NSG
MISTRG
NSG to NSG
MISTRG to MISTRG
1° recipients
2° recipients

Supplementary Figure 6: MSC verification and phenotypic and functional stem cell assessment in serial transplantation.
Related to Fig 5.
a, b Flow cytometric verification of MSC phenotype of primary patient BM-derived **a** human and **b** MISTRG BM-derived murine MSCs (red) compared to isotype control. **c, d** MISTRG BM-derived mesenchymal stromal cells (MSCs) express human in place of murine cytokines. RT-PCR **c** and Q-RT-PCR (in triplicates) **d** detection of human versus murine cytokine expression in 4 patient-derived (P1-4) and representative NSG and MISTRG MSC cultures. (Representative experiment of n=3 independent experiments, statistics represent One way ANOVA with Tukey's multiple comparison calculations; **p<0.01, **** P<0.0001).
e, f Engraftment of clonal MDS-EB-1 (Y016) hematopoiesis. **e** Evidence of paratrabecular $CD34^+$ cells (black arrowheads) in MISTRG but not NSG BM (scale bars 100µM low-power, 20µM high-power magnification); for overall engraftment see Fig. 2. **f** Clonality was determined in representative NSG and MISTRG recipients with engraftment levels > 1% via targeted exome sequencing. Variant allele frequencies (VAFs) in NSG and MISTRG recipients were plotted against the corresponding patient's VAF. Individual mice are represented by symbol shape and mutations are color-coded. Linear regression, Pearson correlations and p-values between patient and xenograft VAF are displayed. **g** Clonality in representative NSG and MISTRG engrafted with MDS-EB-1 (Y014). **h** Enrichment of human cells from NSG and MISTRG BM via depletion of murine $CD45^+$ $Ter119^+$ cells. **i** Percent $CD45^+$ cells before (pre) and after (post) bead-enrichment. **j** Comparison of erythroid lineage engraftment in primary and secondary NSG versus MISTRG recipients of MDS/MPN sample Y013.

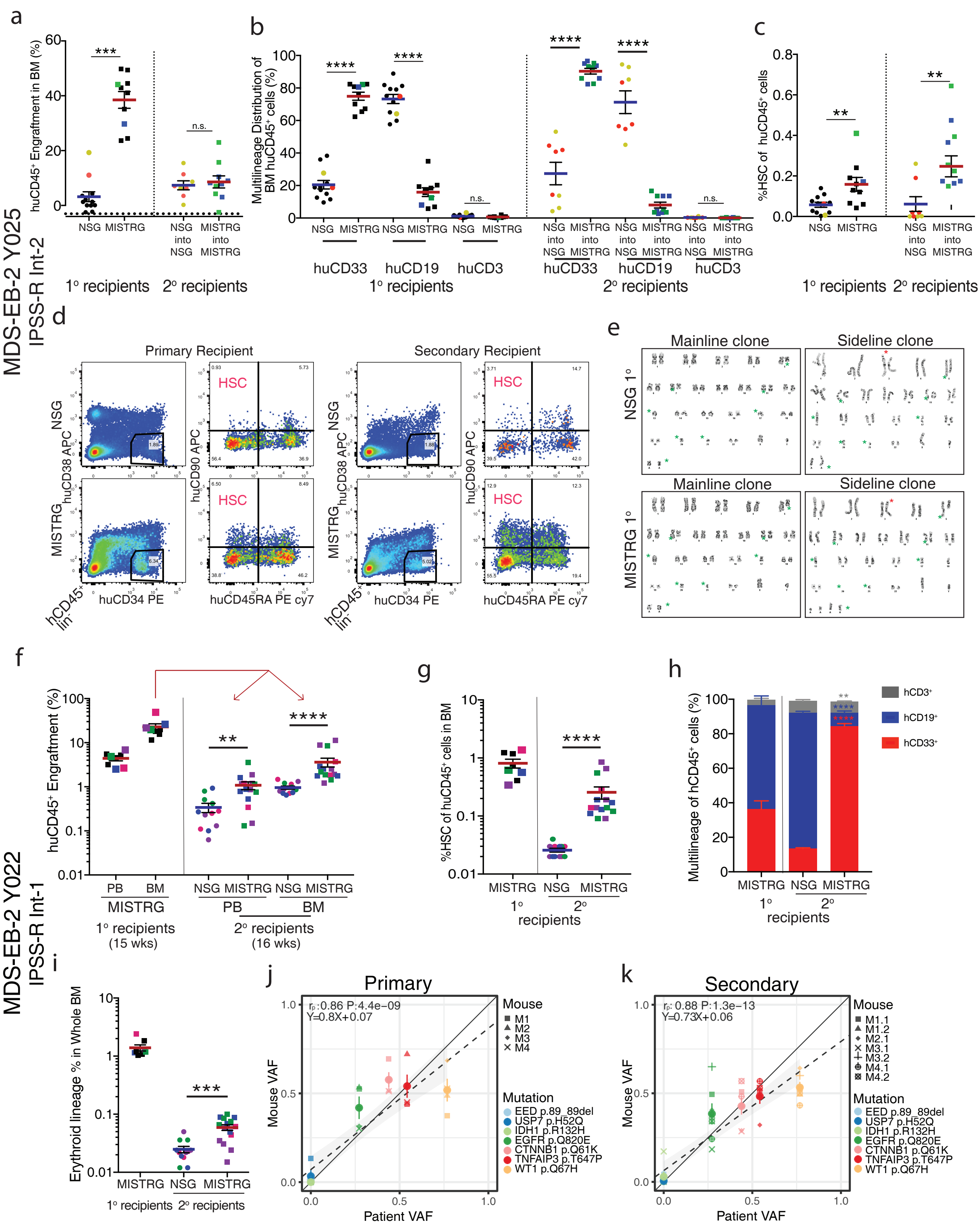

MDS-EB-2 Y025
IPSS-R Int-2
a
huCD45+ Engraftment in BM (%)
***
n.s.
NSG MISTRG
NSG into NSG
MISTRG into MISTRG
1° recipients 2° recipients
b
Multilineage Distribution of BM huCD45+ cells (%)
****
n.s.
huCD33 huCD19 huCD3
1° recipients
2° recipients
c
%HSC of huCD45+ cells
**
d
Primary Recipient
Secondary Recipient
NSG
MISTRG
huCD38 APC
huCD90 APC
HSC
hCD45+ lin-
huCD34 PE
huCD45RA PE cy7
e
Mainline clone
Sideline clone
NSG 1°
MISTRG 1°
MDS-EB-2 Y022
IPSS-R Int-1
f
huCD45+ Engraftment (%)
PB BM
MISTRG
NSG MISTRG NSG MISTRG
PB BM
1° recipients (15 wks)
2° recipients (16 wks)
g
%HSC of huCD45+ cells in BM
MISTRG NSG MISTRG
1° 2°
recipients
h
Multilineage of hCD45+ cells (%)
hCD3+
hCD19+
hCD33+
i
Erythroid lineage % in Whole BM
MISTRG NSG MISTRG
1° recipients 2° recipients
j
Primary
rp:0.86 P:4.4e−09
Y=0.8X+0.07
Mouse VAF
Patient VAF
Mouse
M1
M2
M3
M4
Mutation
EED p.89_89del
USP7 p.H52Q
IDH1 p.R132H
EGFR p.Q820E
CTNNB1 p.Q61K
TNFAIP3 p.T647P
WT1 p.Q67H
k
Secondary
rp: 0.88 P:1.3e−13
Y=0.73X+0.06
M1.1
M1.2
M2.1
M3.1
M3.2
M4.1
M4.2

Supplementary Figure 7: MISTRG faithfully propagate clonal long-term HSC. Serial engraftment, lineage output, and clonal representation of MDS in MISTRG. Related to Fig 5.

a-e Primary and secondary transplantation of high-risk MDS-EB2 (Y025) comparing **a** overall engraftment in BM and **b** multi-lineage representation in BM of primary and secondary NSG and MISTRG recipients. **c** Comparison of phenotypic HSC % in NSG vs. MISTRG primary and secondary recipients. Individual mice are represented by symbols with means ± S.E.M.; symbols for corresponding 1° and 2° recipient mice are color-coded; statistics represent Mann-Whitney test; n.s. not significant, *p<0.05, **p<0.01, ***p<0.001, ****p<0.0001. **d** Representative FACS plots showing HSC gating in primary and secondary NSG and MISTRG recipients. **e** Clonal derivation of primary grafts in NSG and MISTRG mice was verified by cytogenetic analysis replicating the patient's karyotype (see Supplementary Table 1). Composite images of metaphase mainline and sideline clones showing genetic aberrations in NSG (top) and MISTRG (bottom) recipient mice. **f-k** Representation of IPSS intermediate-2 MDS-EB-2 (Y022) engrafted NSG and MISTRG mice. **f** Human $CD45^{+}$ engraftment in MISTRG PB and BM and comparison of engraftment in secondary NSG and MISTRG recipients. **g** Phenotypic HSC (Lin^{-} $CD38^{-}$ $CD34^{+}$ $CD45RA^{-}$ $CD90^{+}$ % of $huCD45^{+}$) of primary MISTRG and secondary NSG and MISTRG recipient mice (Individual mice are represented by symbols with mean ± S.E.M. Corresponding primary and secondary recipient mice are color-coded. Mann Whitney test with *p<0.05, **p<0.01, ***p<0.001, and ****p<0.0001 for aggregate NSG vs. MISTRG). **h** Relative distribution of myeloid CD33+ (red), B-lymphoid CD19+ (blue), and T-lymphoid CD3+ (Grey) cells as % of human CD45+ cells in 1° and 2° NSG vs. MISTRG recipient mice. Stacked bar graphs represent means ± S.E.M. Mann Whitney test; n.s. not significant, *p<0.05, **p<0.01, ***p<0.001, ****p<0.0001, for aggregate NSG vs. MISTRG. **i** Erythroid lineage representation in primary MISTRG and secondary NSG vs MISTRG recipient mice. **j, k** Clonality was determined in representative primary **j** and secondary **k** MISTRG recipients with engraftment levels > 1% via targeted exome sequencing. Variant allele frequencies (VAFs) in primary and secondary recipients were plotted against the corresponding patient's. Individual mice are represented by symbol shape and mutations are color-coded. Linear regression, Pearson correlations and p-values between patient and xenograft VAF are displayed.

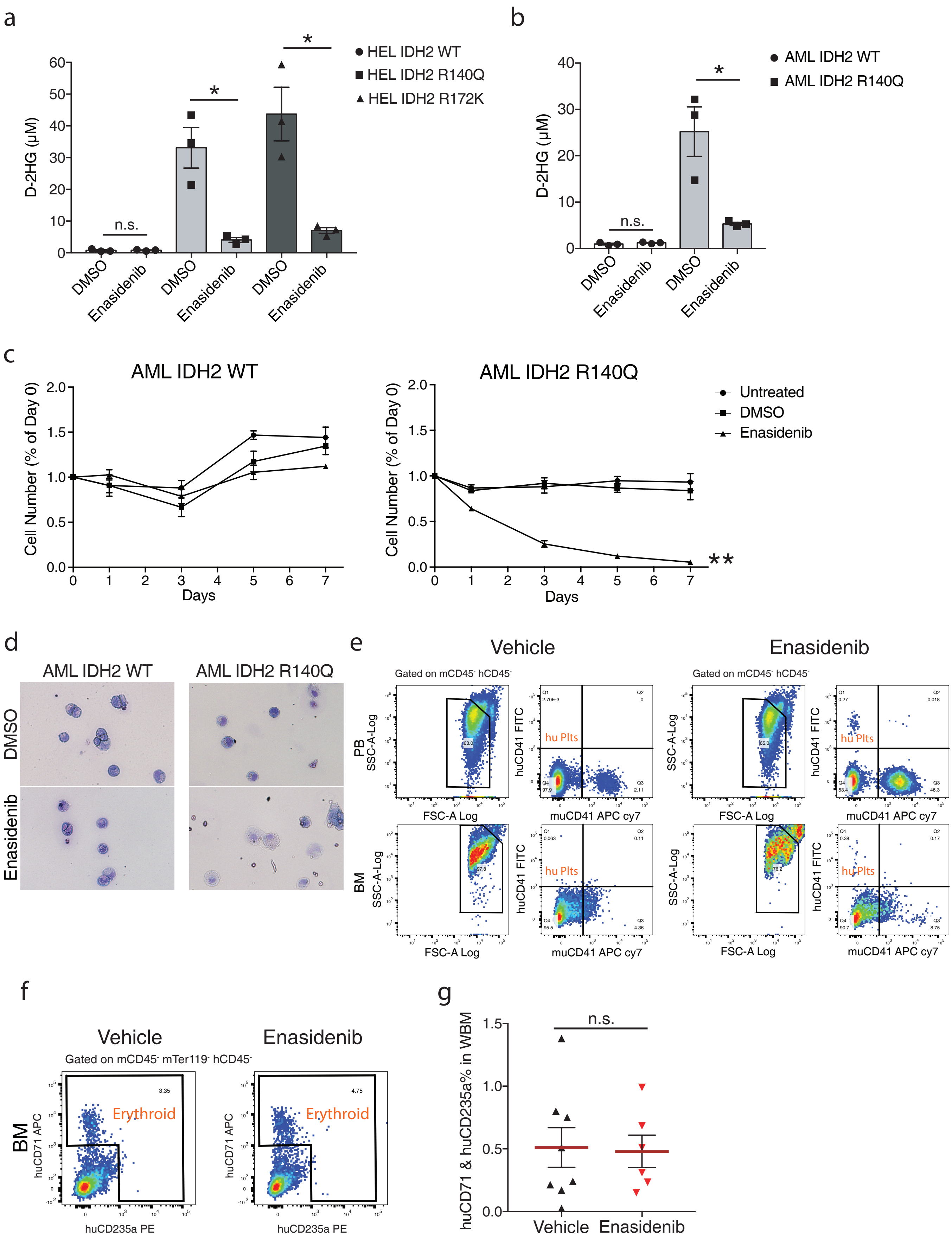
a
D-2HG (µM)
60
50
40
30
20
10
0
n.s.
*
*
DMSO
Enasidenib
DMSO
Enasidenib
DMSO
Enasidenib
HEL IDH2 WT
HEL IDH2 R140Q
HEL IDH2 R172K
b
D-2HG (µM)
40
30
20
10
0
n.s.
*
DMSO
Enasidenib
DMSO
Enasidenib
AML IDH2 WT
AML IDH2 R140Q
c
AML IDH2 WT
AML IDH2 R140Q
Cell Number (% of Day 0)
2.0
1.5
1.0
0.5
0.0
Days
0 1 2 3 4 5 6 7
Untreated
DMSO
Enasidenib
**
d
AML IDH2 WT
AML IDH2 R140Q
DMSO
Enasidenib
e
Vehicle
Enasidenib
Gated on mCD45- hCD45-
PB
BM
SSC-A Log
FSC-A Log
huCD41 FITC
muCD41 APC cy7
hu Plts
f
Vehicle
Enasidenib
Gated on mCD45- mTer119- hCD45-
BM
huCD71 APC
huCD235a PE
Erythroid
3.35
4.75
g
huCD71 & huCD235a% in WBM
1.5
1.0
0.5
0.0
n.s.
Vehicle
Enasidenib

Supplementary Figure 8: Determination of IDH2 inhibition of mutant IDH2 and suppression of 2-HG production by enasidenib.
Related to Fig 6.
a 2-Hydroxyglutarate (2-HG) levels (μM) in supernatants of cultured Human Erythroid Leukemia (HEL) cells transduced with wildtype, R140Q and R172Q mutant IDH2 expressing lentiviral vectors, treated with vehicle or enasidenib 20μM for 72 hours (student t-test, IDH2 WT n.s., R140Q and R172K * $p < 0.05$, n=3). **b** 2-HG levels in supernatants of cultured primary IDH2 WT (Y031) and R140Q mutant (Y029) leukemia cells treated *in vitro* with vehicle or enasidenib 20μM for 72 hours (student t-test, AML IDH2 WT n.s., R140Q *$p < 0.05$, n=3). **c** Relative cell number in untreated versus vehicle (DMSO) versus enasidenib treated IDH2 WT (Y031, left) and R140Q mutant (Y029, right) primary AML (student t-test, IDH2 WT n.s., R140Q *$p<0.05$). **d** Cytospins of DMSO (top) versus enasidenib (bottom) treated IDH2 WT (left) versus R140Q mutant (right) primary AML. Enasidenib causes differentiation of leukemic blasts. **e** Flow analysis scheme for $huCD41^{+}$ expression in PB and BM from vehicle and enasidenib treated MISTRG mice. **f** Flow analysis scheme for $huCD71^{bright/+}$ and $huCD235^{+}$ expression in BM from vehicle and enasidenib treated MISTRG mice. **g** Quantitation of $huCD71^{bright/+}$ and $huCD235^{+}$ expression in BM from vehicle (n=8) and enasidenib (n=6) treated MISTRG mice.

Abbreviations

PB peripheral blood, BM bone marrow, IHC immunohistochemistry, H&E hematoxylin&eosin, S.E.M. Standard Error of the Mean.
MDS-MLD Myelodysplastic syndrome with multilineage dysplasia, MDS-RS-SLD Myelodysplastic syndrome with ring sideroblasts *with single lineage dysplasia, MDS-EB Myelodysplastic syndrome* with excess blasts.
MSCs Mesenchymal stromal cells, HSC Hematopoietic stem cells, M-CSF macrophage colony stimulating factor, GM-CSF granulocyte-macrophage colony stimulating factor, THPO Thrombopoietin.

Supplementary Table 1 Sample information

Patient ID	Enrichment	Total cell ## research (x10e6)	Sample type	Cell ## pre selection (x10e6)	Cell ## post selection (x10e6)	Cell ## injected/ recipient	Analysis weeks post transplant	WHO 2016	IPSS-R	Age	Sex	Blast %	Karyotype	Clinical Sequencing Mutation
Y001	CD34+ +OKT3	141	BM	91.2	3.45	157000	17	n/a	n/a	25	M	n.i.	NK	n/a
Y002	CD34+ +OKT3	320	BM	160	1.78	178000	10.71	n/a	n/a	59	M	n.i.	NK	n/a
Y003	CD34+ +OKT3	945	BM	756	5.19	247000	13.14	n/a	n/a	56	M	n.i.	NK	n/a
Y004	CD34+ +OKT3	25	BM	8.33	0.47	31000	30.29	MDS with isolated del(5q)	Int-1	69	M	1%	46,XY,del(5)(q13q33)[13]/46,XY[7]	n/a
Y005	CD34+ +OKT3	34	BM	27.2	0.65	50000	22.43	MDS-MLD	Int-1	82	M	3%	46,X,-Y,del(7)(q22q36)[13]/ 46,X,-Y[7]	n/a
Y006	CD34+ +OKT3	73	BM	20.8	0.8	100000	17	MDS-MLD	Int-1	83	M	2%	46,xy	n/a
Y007	CD34+ +OKT3	134	BM	53.6	1.68	120000	21	MDS-RS-SLD	low	80	F	2%	46, XX[20]	SF3B1,TET2, KIT
Y008	CD34+ +OKT3	27.5	BM	13.7	0.875	125000	21	MDS-RS-MLD	low	81	M	3%	45,X,-Y[16]/46,XY[4]	SF3B1+, TET2+
Y009	CD34+ +OKT3	68.4	BM	68.4	0.75	150000	15.14	MDS-MLD	Int-2	89	M	1%	47,XY,+19,del(20)(q11.2q13.3),48,idem,+8[14]/96,idemx2,+8,+8[2]/46,XY[4]	n/a
Y010	CD34+ +OKT3	70	BM	30	2.59	199000	21	MDS/MPN-RS-T	low	69	F	1%	46,XX,del(5)(q15q33)[2]/46, XX[13]	SF3B1+, TET2+
Y011	CD34+ +OKT3	2208	BM	1320	4.14	230000	22.43	MDS-RS-SLD	Int-1	72	M	1%	46,XY,+1,dic(1;15)(p11;p11)[6]/46,XY[14]	n/a
Y012	CD3- +OKT3	228	BM	228	2.2	360000	13.29	MDS-MLD	Int-1	84	F	3%	46,XX[20]	n/a
Y013	CD34+ +OKT3	1065	BM	959	8.13	189000	25.71	MDS-EB-1	low	62	M	3%	46,XY[20]	n/a
Y014	CD34+ +OKT3	800	BM	480	3.8	222000	14.29	MDS-EB-1	Int-1	57	F	6%	46,XX[20]	n/a
Y015	CD34+ +OKT3	320	BM	160	5.04	229000	13.29	MDS-EB-1	Int-1	64	M	8%	46,XY[20]	n/a
Y016	CD3- +OKT3	540	BM	120	3.7	610000	13.29	MDS-EB-1	Int-1	22	F	8%	47,XX,+8[4],XX[11]	n.d.
Y017	CD34+ +OKT3	3	BM	3	1.83	83000	12.29	MDS-EB-2	High	70	F	18%	42,XX,del(5)(q31q35),-7,-12,-16,add(19)(p13.3), der(20)t(20;21)(q11.2;q11.2),-21[15]	n/a
Y018	CD34+ +OKT3	50	BM	25	1.53	85000	20.71	MDS-EB-2	High	72	F	11%	46,XX,del(7)(q32)[5]/46,XX[10].ish del(7)(q32)(RELN+,TES+)[1]	IDH2 R140Q, SRSF2 P95R, CEBPA ins p.Pro197His
Y019*	CD3- +OKT3	45	BM	45	1.61	115000	14.86	MDS-EB-1	Int-2	59	F	5%	46,XX[20]	DNMT3A I1310S
Y020	CD34+	n/a	BM	n/a	n/a	115000	14	MDS-EB-2	high	82	M	14%	45,XY,del(3)(p21),del(5)(q13.3q34),-14,-18,der(20)t(14;20)(q11;q11.2)dup(20)(q11.21q11.22)del(20)(Q11.22q31.2),+mar[cp8]/46XY[7]	n/a
Y021	CD34+ +OKT3	157.6	BM	67.5	2.1	131000	15.57	MDS-EB-2	High	59	M	10%	46,XY,der(15)t(1;15)(q12;p12)[7]/46, XY, der(21)t(1;21)(q12;p12)[3]/ 46,XY, der(13)t(1;13)(q12;p12)[2]/46,XY, der(14)t(1;14)(q12;p12)[2]/46,XY[6] nuc ish(PBX1x3, TCR3x2)[64/200]	IDH2 R140Q, SRSF2 P95L
Y022	CD34+ +OKT3	417	BM	292	4.62	165000	15.14	MDS-EB-2	Int-1	76	M	6%	46,XY,del(2)(p21p23)[13]/46,XY[2]	n/a
Y023	CD34+ +OKT3	69.5	BM	46	4.22	192000	13.14	MDS-EB-2	Int-2	62	F	15%	46,XX[20]	No mutation detected
Y024	CD34+ +OKT3	23	BM	2.5	1.6	200000	29.29	MDS-EB-2	Int-2	72	M	18%	47,XY,+8[15]/46,XY[1]	NRAS, CSF3R, MPL
Y025	CD34+ +OKT3	200	BM	200	6.1	275000	16.14	MDS-EB-2	Int-2	64	M	10%	44~46,XY,del(5)(q13q33),der(7)t(7;17)(q11.2;q11.2),-13,-17,-20,-21,+2~4mar[6]/44,idem,add(3)(p25),add(11)(q23)[6]/46,XY[3]	n/a
Y026	CD3- +OKT3	46	BM	23	1.7	340000	13.29	MDS-EB-2	Int-2	24	F	15-19%	NK	n/a
Y027	CD34+ +OKT3	219	BM	131.4	5.39	317000	23	AML	n/a	91	F	19%	46,XX,-3,del(5)(q11.2),-8,der(12)t(3;12)(q22;p1),-17,i(17)(q10)	TP53 Level 2 variant
Y028*	CD3- +OKT3	40	PB	20	6.6	550000	23.86	sAML	n/a	59	F	62%	NK	DNMT3a I1310S, IDH1 R132C
Y029	CD3- +OKT3	1220	PB	610	310	1000000	12.71	sAML	n/a	57	M	35%	46,XY[20]	n/a
Y030	CD34+ +OKT3	428	BM	283	16.8	1400000	9.57	AML	n/a	55	M	76%	NK	n/a

Y031	CD34+ +OKT3	2559	BM	255.9	10.5	875000	18.71	AML	n/a	76	M	85%	NK	FLT3-ITD, SRSF2 missense, JAK-2 V617F

*** same patient with different disease stage**

Targeted Exome Sequencing Result

									Patient Variant Allele Frequency	Xenograft Variant Allele Frequency		
Patient ID: Y006	Chr	Start	End	Ref	Alt	Gene Name	Amino Acid Change	Mutation Type	Y006	MISTRG 1	MISTRG 2	MISTRG 3
	Chr 18	42531907	42531907	G	A	SETBP1	D868N	missense	0.2265	0.0488	0.0461	0.0441
	Chr 18	42531913	42531913	G	A	SETBP1	G870S	missense	0.1657	0.0245	0.0321	0.0203
	Chr 21	44514777	44514777	T	C	U2AF1	Q84R	missense	0.3988	0.4737	0.4973	0.4692
	Chr 7	55221800	55221800	G	A	EGFR	E282K	missense	0.3594	0.3550	0.4215	0.3635
	Chr 19	17945696	17945696	C	T	JAK3	V722I	missense	0.4971	0.5048	0.4938	0.5274

Patient ID: Y011	Chr	Start	End	Ref	Alt	Gene Name	Amino Acid Change	Mutation Type	Y011	MISTRG 1	MISTRG 2
	Chr 1	36932275	36932275	C	T	CSF3R	D732N	missense	0.5200	0.5926	0.5333
	Chr 13	32912750	32912750	G	T	BRCA2	D1420Y	missense	0.6000	0.5011	0.4208
	Chr 13	32972606	32972608	CTC	-	BRCA2	3319_3320del	missense	0.6368	0.6419	0.6203
	Chr 2	198267489	198267489	T	C	SF3B1	P623R	missense	0.0261	0.0401	0.1236
	Chr 7	151932990	151932990	C	T	KMT2C	R894Q	missense	0.3442	0.3645	0.3355

Patient ID: Y012	Chr	Start	End	Ref	Alt	Gene Name	Amino Acid Change	Mutation Type	Y012	MISTRG 1	MISTRG 2
	Chr 21	44514777	44514777	T	G	U2AF1	Q84P	missense	0.3177	0.6899	0.4384
	Chr 21	36252853	36252853	C	T	RUNX1	ss 973 C>T	splicing	0.3361	1.0000	0.4951
	Chr 7	151859899	151859899	G	A	KMT2C	S3588L	missense	0.5065	0.3235	0.4893
	Chr 12	49444975	49444975	A	G	KMT2D	S831P	missense	0.7327	0.2581	0.5145

									Patient Variant Allele Frequency	Primary Recipients						Secondary Recipients			
Patient ID: Y013	Chr	Start	End	Ref	Alt	Gene Name	Amino Acid Change	Mutation Type	Y013	MISTRG 1	MISTRG 2	MISTRG 3	MISTRG 4	NSG 1	NSG 2	MISTRG 4.1	MISTRG 4.2	NSG 2.1	NSG 2.2
	Chr 2	25965532	25965532	C	G	ASXL2	C1225S	missense	0.4640	0.3773	0.7075	0.4361	0.0827	0.4234	0.2975	0.3275	0.3445	0.3361	0.3565
	Chr 17	74732959	74732959	G	C	SRSF2	P95R	missense	0.2105	0.4203	0.5592	0.6029	0.6203	0.5057	0.3228	0.4890	0.4870	0.6099	0.3953
	Chr 10	112360195	112360195	A	T	SMC3	ss 2912 A>T	splicing	0.4436	0.6916	0.4000	0.4320	0.3640	0.4701	0.5762	0.5176	0.5110	0.4991	0.5805
	Chr 9	93626937	93626937	A	C	SYK	I262L	missense	0.5436	0.5325	0.5671	0.6123	0.3955	0.5183	0.6121	0.4916	0.4769	0.5782	0.4204
	Chr 1	145281677	145281677	T	C	NOTCH2NL	F203L	missense	0.1375	0.1357	0.1317	0.1314	0.1204	0.1245	0.0896	0.1408	0.1290	0.1034	0.1399
	Chr 13	32912582	32912582	A	C	BRCA2	I1364L	missense	0.5138	0.4608	0.3945	0.4122	0.3313	0.4790	0.2797	0.4517	0.4596	0.5353	0.4020
	Chr 7	151962285	151962285	T	A	KMT2C	D341V	missense	0.0707	0.0762	0.0260	0.0616	0.0838	0.0212	0.1251	0.0194	0.0257	0.0152	0.0325
	Chr 7	128846326	128846326	G	A	SMO	G388R	missense	0.5431	0.5485	0.4009	0.5825	0.5117	0.5168	0.5092	0.5034	0.5203	0.5943	0.5247
	Chr 10	70332591	70332591	C	T	TET1	L166F	missense	0.4449	0.4204	0.4554	0.4432	0.2291	0.4750	0.5275	0.4520	0.4545	0.4583	0.4383
	Chr 7	55249160	55249160	C	G	EGFR	Q820E	missense	0.0000	0.0007	0.2381	0.2797	0.3323	0.4423	0.2614	0.3481	0.3350	0.3934	0.0467

Patient ID: Y014	Chr	Start	End	Ref	Alt	Gene Name	Amino Acid Change	Mutation Type	Y014	MISTRG 1	MISTRG 2	NSG
	Chr 17	74732959	74732959	G	A	SRSF2	P95L	missense	0.2767	0.3940	0.2572	0.4736
	Chr 20	57428948	57428948	G	C	GNAS	A210P	missense	0.2139	0.0971	0.2000	0.2462
	Chr X	123200043	123200043	G	T	STAG2	K705N	missense	0.0169	0.0092	0.0161	0.1350
	Chr 20	31022439	31022443	GGAGG	-	ASXL1	642_643del	missense	0.1833	0.2844	0.4531	0.5099

Patient ID: Y016	Chr	Start	End	Ref	Alt	Gene Name	Amino Acid Change	Mutation Type	Y016	MISTRG 1	MISTRG 2	NSG
	Chr 7	124499090	124499090	C	T	POT1	S208N	missense	0.5632	0.4884	0.4113	0.0385
	Chr 12	49444427	49444427	T	C	KMT2D	I982V	missense	0.5158	0.5074	0.4995	0.4326
	Chr 11	32417947	32417947	G	C	WT1	R369G	missense	0.0417	0.0000	0.0014	0.0000
	Chr 7	151932990	151932990	C	T	KMT2C	R894Q	missense	0.2993	0.2813	0.3133	0.3128
	Chr 16	3641124	3641124	G	T	SLX4	H839N	missense	0.0011	0.0074	0.0020	0.1243

									Patient Variant Allele Frequency	Primary Recipients					
Patient ID: Y019	Chr	Start	End	Ref	Alt	Gene Name	Amino Acid Change	Mutation Type	Y019	MISTRG 1	MISTRG 2	MISTRG 3	MISTRG 4	MISTRG 5	MISTRG 6
	Chr 22	41562607	41562607	G	C	EP300	V1271L	missense	0.4444	0.5347	0.8710	0.3000	0.4725	0.4109	0.5258
	Chr X	39921553	39921553	-	C	BCOR	Q1389fs	nonsense	0.0581	0.1907	0.0726	0.2043	0.3684	0.2104	0.0021
	Chr 2	209113113	209113113	G	A	IDH1	R132C	missense	0.0083	0.2907	0.0000	0.0000	0.3292	0.1804	0.0002

	Chr 2	25470545	25470545	A	C	DNMT3A	I310S	missense	0.2000	0.4583	1.0000	0.5000	0.8796	0.5651	0.0087
	Chr 1	115258748	115258748	C	T	NRAS	G12S	missense	0.0000	0.0000	0.0000	0.0000	0.0004	0.0006	0.0000
	Chr X	129150016	129150016	C	T	BCORL1	R1090X	missense	0.0391	0.1270	0.0000	0.0804	0.2715	0.2691	0.0015
	Chr 21	11058316	11058316	G	T	BAGE3	H42N	missense	0.2759	0.2660	0.0000	0.4348	0.2588	0.1206	0.1048

									Patient Variant Allele Frequency	Vehicle treated			Enasidenib treated	
Patient ID: Y021	Chr	Start	End	Ref	Alt	Gene Name	ıino Acid Char	Mutation Type	Y021	MISTRG Vehicle 1	MISTRG Vehicle 2	MISTRG Vehicle 3	MISTRG Enasidenib 1	MISTRG Enasidenib 2
	Chr 17	1560006	1560006	A	-	PRPF8	L1852fs	nonsense	0.2859	0.3494	0.2923	0.1619	0.1615	0.2284
	Chr 7	151970835	151970835	T	C	KMT2C	I323V	missense	0.3391	0.4332	0.1644	0.3375	0.3162	0.2428
	Chr 15	90631934	90631934	C	T	IDH2	R140Q	missense	0.3814	0.4534	0.1705	0.0619	0.4354	0.0368
	Chr X	123171406	123171406	C	A	STAG2	Y106X	missense	0.8073	1.0000	0.5197	0.0881	0.7273	0.0000
	Chr 17	74732959	74732959	G	A	SRSF2	P95L	missense	0.2594	0.4379	0.1927	0.0329	0.2499	0.0839
	Chr 17	30325922	30325922	G	T	SUZ12	G707V	missense	0.0026	0.0006	0.1218	0.0012	0.0008	0.0010
	Chr 9	93626937	93626937	A	C	SYK	I262L	missense	0.5145	0.3744	0.4346	0.4526	0.5474	0.4467
	Chr 7	151860210	151860210	C	A	KMT2C	Q3484H	missense	0.1714	0.0009	0.0004	0.0005	0.0004	0.0000

									Patient Variant Allele Frequency	Primary	Secondary		Primary	Secondary	Primary	Secondary		Primary	Secondary	
Patient ID: Y022	Chr	Start	End	Ref	Alt	Gene Name	Amino Acid Change	Mutation Type	Y022	MISTRG 1	MISTRG 1.1	MISTRG 1.2	MISTRG 2	MISTRG 2.1	MISTRG 3	MISTRG 3.1	MISTRG 3.2	MISTRG 4	MISTRG 4.1	MISTRG 4.2
	Chr 7	55249160	55249160	C	G	EGFR	Q820E	missense	0.2720	0.5251	0.4947	0.3723	0.5331	0.4063	0.3161	0.1837	0.6500	0.3016	0.2418	0.3422
	Chr 3	41266184	41266184	C	A	CTNNB1	Q61K	missense	0.4391	0.6942	0.5093	0.4299	0.5150	0.2901	0.5855	0.2857	0.0000	0.5126	0.4819	0.5690
	Chr 11	32456691	32456691	C	A	WT1	Q67H	missense	0.7681	0.3747	0.5314	0.4928	0.4869	0.6421	0.6850	0.0000	0.6000	0.5273	0.4318	0.5000
	Chr 11	85961490	85961498	GGTATGTGC	-	EED	89_89del	missense	0.0000	0.0000	0.0431	0.0139	0.0374	0.0526	0.0091	0.0000	0.0000	0.0089	0.0000	0.0000
	Chr 16	9024178	9024178	G	T	USP7	H52Q	missense	0.0000	0.1343	0.0000	0.0000	0.0000	0.0269	0.0000	0.0000	0.0000	0.0002	0.0000	0.0000
	Chr 6	138201240	138201240	A	C	TNFAIP3	T647P	missense	0.5422	0.4389	0.5145	0.4815	0.7207	0.3211	0.5532	0.0000	0.0000	0.4493	0.5684	0.5305
	Chr 2	209113112	209113112	C	T	IDH1	R132H	missense	0.0000	0.0001	0.0019	0.0014	0.0000	0.0000	0.0010	0.1719	0.0000	0.0000	0.0000	0.0000

Patient ID: Y023	Chr	Start	End	Ref	Alt	Gene Name	Amino Acid Change	Mutation Type	Y023	MISTRG 1	MISTRG 2
	Chr 8	61654298	61654298	T	A	CHD7	S103T	missense	0.4774	0.4494	0.4721
	Chr 16	14041570	14041570	T	C	ERCC4	I706T	missense	0.4442	0.4605	0.3859

Patient ID: Y027	Chr	Start	End	Ref	Alt	Gene Name	Amino Acid Change	Mutation Type	Y027	MISTRG 1	MISTRG 2
	Chr 16	3831230	3831230	G	T	CREBBP	L551I	missense	0.4752	0.4935	0.4612
	Chr 17	7578272	7578272	G	T	TP53	H154N	missense	0.5838	0.9268	0.9322
	Chr 22	41513455	41513455	G	A	EP300	S120N	missense	0.4718	0.5072	0.1640
	Chr X	70462106	70462106	T	A	ZMYM3	Q1239L	missense	0.3823	0.4933	0.3750
	Chr 2	74273609	74273609	G	T	TET3	A189S	missense	0.0000	0.0147	0.2133

Patient ID: Y028	Chr	Start	End	Ref	Alt	Gene Name	Amino Acid Change	Mutation Type	Y028	MISTRG 1	MISTRG 2
	Chr 22	41562607	41562607	G	C	EP300	V1271L	missense	0.7754	0.4970	0.5362
	Chr X	39921553	39921553	-	C	BCOR	Q1389fs	nonsense	0.3741	0.4988	0.4916
	Chr 2	209113113	209113113	G	A	IDH1	R132C	missense	0.2419	0.4156	0.3426
	Chr 2	25470545	25470545	A	C	DNMT3A	I310S	missense	0.8203	0.9952	0.9967
	Chr 1	115258748	115258748	C	T	NRAS	G12S	missense	0.0414	0.4886	0.4597
	Chr X	129150016	129150016	C	T	BCORL1	R1090X	missense	0.2562	0.3955	0.3963
	Chr 21	11058316	11058316	G	T	BAGE3	H42N	missense	0.0039	0.0994	0.0935

Suppplementary Table 3 Antibody Information, Flow cytometry

Panel	Antibody name	Clone	Maker	Dilution
Panel A2	PacificBlue muCD45	30-F11	Biolegend	1:500
	PerCP/Cy5.5 huCD45	HI30	Biolegend	1:300
	PE huCD34	561	Biolegend	1:100
	FITC huCD38	HIT2	Thermo Fisher Scientific	1:100
	PE/Cy7 huCD45RA	HI100	Biolegend	1:500
	APC huCD90	5E10	Biolegend	1:100
	Biotin huCD2	RPA-2.10	Biolegend	1:200
	Biotin huCD3	HIT3a	Biolegend	1:200
	Biotin huCD4	OKT4	Biolegend	1:200
	Biotin huCD7	124-1D1	Thermo Fisher Scientific	1:200
	Biotin huCD8	RPA-T8	Biolegend	1:200
	Biotin huCD10	SN5c	Thermo Fisher Scientific	1:200
	Biotin huCD11b	ICRF44	Thermo Fisher Scientific	1:200
	Biotin huCD14	63D3	Biolegend	1:200
	Biotin huCD19	HIB19	Thermo Fisher Scientific	1:200
	Biotin huCD20	2H7	Thermo Fisher Scientific	1:200
	Biotin huCD56	5.1H11	Biolegend	1:200
	Biotin huCD235ab	HIR2	Biolegend	1:200
	SA APCCy7 Streptavidin		Biolegend	1:300
Panel B3				
	PacificBlue muCD45	30-F11	Biolegend	1:500
	PerCP/Cy5.5 huCD45	HI30	Biolegend	1:300
	PE huCD34	561	Biolegend	1:100
	FITC huCD3	OKT3	Biolegend	1:50
	PE/Cy7 huCD19	HIB19	Biolegend	1:200
	Biotin huCD335	9E2	Biolegend	1:500
	SA APC/Cy7 Streptavidin		Biolegend	1:300
	APC huCD33 (1:100)	WM53	Biolegend	1:100
Panel C2				
	PacificBlue muCD45	30-F11	Biolegend	1:500
	PerCP/Cy5.5 huCD45	HI30	Biolegend	1:300
	FITC huCD64	10.1	Biolegend	1:100
	PE huCD34	561	Biolegend	1:100
	PE/Cy7 huCD10	HI10a	Biolegend	1:200
	APC huCD33	WM53	Biolegend	1:100
	APC/Cy7 huCD14	63D3	Biolegend	1:100
Panel D2				
	PacificBlue muCD45	30-F11	Biolegend	1:500
	PerCP/Cy5.5 huCD45	HI30	Biolegend	1:300
	FITC huCD16	3G8	Biolegend	1:100
	PE huCD13	WM15	Biolegend	1:100
	PE/Cy7 huCD15	W6D3	Biolegend	1:200
	APC huCD11b	ICRF44	Biolegend	1:200
	APC/Cy7 huCD14	63D3	Biolegend	1:100
Panel E2				
	PacificBlue muCD45	30-F11	Biolegend	1:500

	PerCP/Cy5.5 huCD45	HI30	Biolegend	1:300
	FITC huCD41	HIP8	Biolegend	1:100
	PE huCD235	HI264	Biolegend	1:500
	APC huCD71	CY1G4	Biolegend	1:100
	PE/Cy7 muTer119	Ter-119	Biolegend	1:300
human MSC panel				
	PE huCD44	BJ18	Biolegend	1:500
	APC huCD90	5E10	Biolegend	1:100
	PerCP/Cy5.5 huCD45	HI30	Biolegend	1:500
	FITC huCD29	TS2/16	Thermo Fisher Scientific	1:100
	eFluor450 huCD105	SN6	Thermo Fisher Scientific	1:100
mouse MSC panel				
	PacificBlue MuCD45	30-F11	Biolegend	1:500
	FITC muCD29	HMβ1-1	Biolegend	1:100
	APC muCD44	IM7	Biolegend	1:500
	PerCP/Cy5.5 muCD90	30-H12	Biolegend	1:100
	PE muSca-1	D7	Biolegend	1:100
	Purified Rat Anti-Mouse CD16/CD32 (Mouse BD Fc Block™)	2.4G2	BD Biosciences	1:100
Murine cells depletion				
	Biotin anti-mouse CD45	30-F11	Biolegend	
	Biotin anti-mouse TER-119/Erythroid Cells Antibody	TER-119	Biolegend	
	Streptavidin Particles Plus - DM antibody		BD Biosciences	
Panel HSC				
	APC/Cy7 mCD45	30-F11	Biolegend	1:300
	APC/Cy7 mTer119	Ter-119	Biolegend	1:300
	BV510 hCD45	HI30	Biolegend	1:100
	APC huCD135	BV10A4H2	Biolegend	1:100
	BV421 huCD38	HIT2	Biolegend	1:100
	AF488 huCD45RA	HI100	Biolegend	1:300
	PerCP/Cy5.5 huCD90	5E10	Biolegend	1:100
	PE huCD34	561	Biolegend	1:100
	PE/Cy7 huCD10	HI10a	Biolegend	1:200
	PE/Dazzle594 huCD123	6H6	Biolegend	1:100

Supplementary Table 4 Antibody information, Histology

Antibody name	Reactivity	Clone	Maker
Anti-Glycophorin A antibody	Human	EPR8200	abcam
CD68/Macrophage Marker Ab-4, Mouse Monoclonal Antibody	Human	PG-M1	Thermo Fisher Scientific
CD34 Class II	Human	QBEnd 10	Dako
CD61, Platelet Glycoprotein IIIa	Human	Y2/51	Dako
CD45, Leucocyte Common Antigen	Human	PD7/26 + 2B11	Dako
CD15 COCKTAIL	Human	MMA + BY87	Biocare Medical
Von Willebrand Factor	Human	Polyclonal	Dako
F4/80 Monoclonal Antibody	Human	SP115	Thermo Fisher Scientific
MPO	Human	polyclonal	Dako
Reticulin	Standard Chemical Stains		
Prussian Blue Iron Stain	Standard Chemical Stains		

Supplementary Table 5 PCR PRIMER SEQUENCES

Cytokines, Q-PCR

Gene Name	Primer	Product length (BP)
Murine GM-CSF forward	CAG GGT CTA CGG GGC AAT TT	100
Murine GM-CSF Reverse	CAC AGT CCG TTT CCG GAG TT	
Murine Thpo forward	TAA CTC TGT CCA GCC CCG TA	185
Murine Thpo Reverse	GCT CTG TTC CGT CTG GGT TT	
Murine M-CSF forward	AGT ATT GCC AAG GAG GTG TCA G	107
Murine M-CSF Reverse	ATC TGG CAT GAA GTC TCC ATT T	
Human GM-CSF forward	AAA TGT TTG ACC TCC AGG AGC C	134
Human GM-CSF Reverse	AGG GCA GTG CTG CT GTA G	
Human Thpo forward	GGTTCACCCTTTGCCTACACC	77
Human Thpo Reverse	CCT CCA TCT GGG TTT TCC ATT C	
Human M-CSF forward	TCC AGC CAA GAT GTG GTG AC	153
Human M-CSF Reverse	AGT TCC CTC AGA GTC CTC CC	
18S forward	GGC CCT GTA ATT GGA ATG AGT C	146
18S Reverse	CCA AGA TCC AAC TAC GAG CTT	

PCR Program - for gel visualization	qPCR Program
95°C – 2 minutes , 35 cycles of (95°C – 30 seconds, 60°C – 60 seconds, 72°C – 80 seconds), 72°C – 10 minutes.	94°C – 4 minutes , 30 cycles of (94°C – 30 seconds, 60°C – 30 seconds, 72°C – 45 seconds), 72°C – 5 minutes.

IDH2-WT-FLAG amplification forward	TAG CGG CCG CGA ACC GTC AGA TCG CCA CTA
IDH2-WT-FLAG amplification reverse	GGC TCG AGA TGT TAT CAA CCA
R140Q Site-Directed Mutagenesis Primer Forward	CCC CCC AGG ATG TTC TGG ATA GTT CCA TTG G
R140Q Site-Directed Mutagenesis Primer Reverse	CCA ATG GAA CTA TCC AGA ACA TCC TGG GGG G

Supplementary Table 6 Targeted Exome Gene List

**GENE ID**
ABL1
ABL2
AKT1
ALK
APC
ASXL1
ASXL2
ATM
ATRX
AURKA
AURKB
AURKC
BCL2
BCOR
BCORL1
BRAF
CALR
CBL
CBLB
CBLC
CDH1
CDKN2A
CEBPA
CHD7
CREBBP
CSF1R
CSF3R
CTNNB1
CUX1
DICER1
DNMT3A
DOT1L
EED
EGFR
EP300
ERBB2
ERBB4
ETV6
EZH2
FANCA
FANCB
FANCC
FANCD1
FANCD2
FANCE
FANCF
FANCG
FANCI
FANCJ
FANCL
FANCM
FANCN
FANCO
FANCP
FANCQ

FBXW7
FGFR1
FGFR2
FGFR3
FLT3
GATA1
GATA2
GATA2
GATA3
GNA11
GNA13
GNAQ
GNAS
H3F3A
HDAC9
HNRNPA1
HRAS
IDH2
IKZF1
IL7R
JAK1
JAK2
JAK3
KDM4C
KDM6A
KIT
KMT2A
KMT2C
KMT2D
KRAS
MAP2K1
MAP2K2
MAP2K4
MET
MPL
MYD88
NOTCH1
NOTCH2
NOTCH4
NPM1
NRAS
NSD2
PAX5
PDGFRA
PDGFRB
PHF6
PIK3CA
PIK3R1
POT1
PRPF40B
PRPF8
PTEN
PTPN11
RAD21
RET
RHOA
RUNX1
SETBP1

SETD2
SF3A1
SF3B1
SMAD4
SMARCA4
SMC1A
SMC3
SMO
SOCS1
SRC
SRSF2
STAG2
STAT3
STK11
SUZ12
SYK
TET1
TET2
TET3
TNFAIP3
TP53
U2AF1
U2AF2
USP7
VHL
WT1
XPO1
ZMYM3
ZRSR2
